# Supplementary material for: Vascular progenitors generated from tankyrase inhibitor-regulated naïve diabetic human iPSC potentiate efficient revascularization of ischemic retina
Source: Nat Commun. 2020 Mar 5;11:1195. doi: 10.1038/s41467-020-14764-5 (PMC7058090; doi:10.1038/s41467-020-14764-5)

SUPPLEMENTARY INFORMATION

Vascular Progenitors Generated from Tankyrase Inhibitor-Regulated Naïve Diabetic Human iPSC Potentiate Efficient Revascularization of Ischemic Retina

Park *et. al*

Supplementary Figures

Supplementary Figure 1. ***In vitro* multi-lineage directed differentiations of paired isogenic primed *vs.* naïve normal (non-diabetic) hiPSC lines.** Isogenic primed (E8 cultures) vs. naïve-reverted (LIF-3i cultures) hiPSC were differentiated in parallel using established multi-lineage protocols and commercially available kits, as previously described and without any requirement for re-priming [12,13]. Shown are direct comparisons between normal (non-diabetic) isogenic hiPSC lines demonstrating augmented differentiation capacities to all three germ layers and robust/improved capacity for terminal differentiation. (**a**) Neuro-ectodermal differentiation of isogenic comparisons of primed *vs*. naïve hPSC lines performed as described with PSC neural induction medium [12] (Thermo Fisher Scientific; *n*=6, three normal fibroblast-hiPSC lines (circles; C1.2, C2, 7ta) and three normal cord blood (CB)-derived hiPSC lines (triangles; E5C3, E5C1, LZ6+10); see **Table S1**). Shown are flow cytometry analysis of differentiation cultures for protein expressions of % Nestin$^+$SOX1$^+$ neural progenitor cells determined by flow cytometry at day 10 neural differentiation, as described [12,13]. (**b**) Representative images of Tuj1-expressing terminally-differentiated cells from a primed (E8 culture) vs naïve (LIF-3i culture) CB-iPSC line (E5C3) after 5 weeks of neural differentiation, performed as described [12,13], and demonstrating higher incidence of extended Tuj1$^+$ neurites elongated over 3 mm in N-CB-iPSC relative to primed CB-iPSC. (**c**) Definitive endodermal differentiation of isogenic primed vs naïve-hiPSC (*n*=6; hiPSC lines as above (**Table S1**), each individual line represented by a different color, with mean and SEM shown). Differentiations were performed as described with STEMdiff definitive endoderm kit and STEMdiff APEL medium (StemCell Technologies) [12]**.** Shown are % differentiated cells expressing FOXA2$^+$ endodermal progenitor cells.

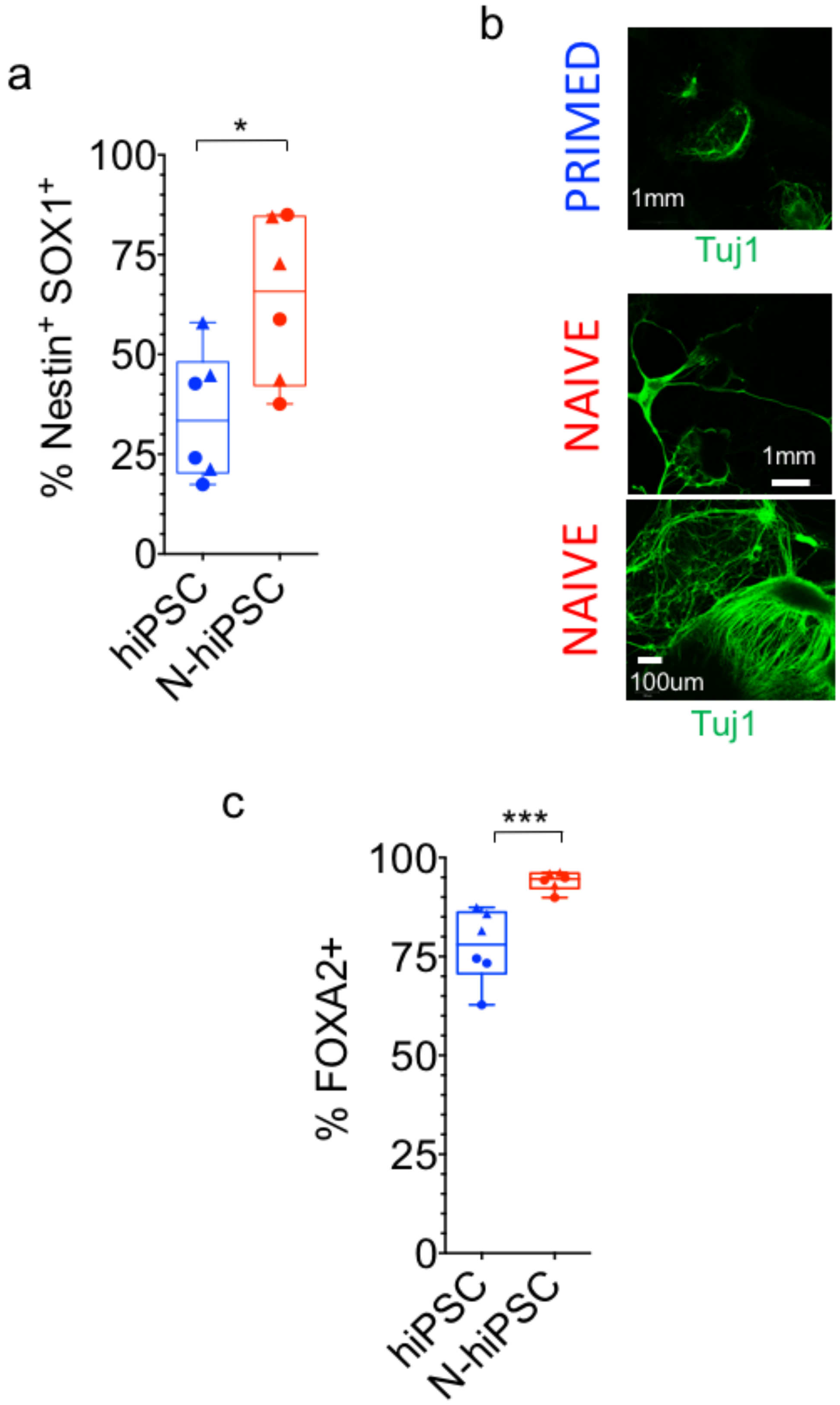
a
*
100
75
50
25
0
% Nestin+ SOX1+
hiPSC
N-hiPSC
b
PRIMED
1mm
Tuj1
NAIVE
1mm
NAIVE
100um
Tuj1
c
***
100
75
50
25
0
% FOXA2+
hiPSC
N-hiPSC

Supplementary Figure 2. **Non-integrated episomal reprogramming of type I diabetic skin fibroblasts into conventional DhiPSC lines, and subsequent naïve reversion into N-DhiPSC with the LIF-3i culture system**. (**a**) Scheme of timeline of reprogramming of diabetic skin fibroblasts for the generation of conventional, primed DhiPSC. FGM: fibroblast growth medium, ES: ES medium, AA: ascorbic acid, CHIR99021: GSK3-β inhibitor, E8: essential 8 medium. (**b**[a]) typical morphology of conventional primed DhiPSC cultured in E8 medium on vitronectin-coated plates. (**b**[b]) teratoma formation in NOG mice from primed DhiPSC line (E1CA1) generated well-developed three germ layer organoid structures. (**c**) Flow cytometry analysis of conventional DhiPSC lines demonstrated >95% $SSEA4^{+}TRA1\text{-}81^{+}$ expressions. (**d**) Scheme of timeline of naïve reversion (LIF-5i/LIF-3i) of primed, conventional DhiPSC into N-DhiPSC, performed as described [12]. (**e**) typical dome-shaped colonies (left, [a,c]) in LIF-3i naïve cultures of N-DhiPSC lines (E1C1, E1CA2 – see **Table S1**) and their normal G-banded karyotypes (right, [b,d]) following 3-10 passages in naïve (LIF-3i) conditions. (**f**) Representative teratoma H&E sections from two N-DhiPSC lines (E1CA2 and E1C1). Shown are ectodermal neural rosette (Ect), endodermal epithelial gut (End), and mesodermal cartilage (Meso) structures. Scale bar = 100 um.

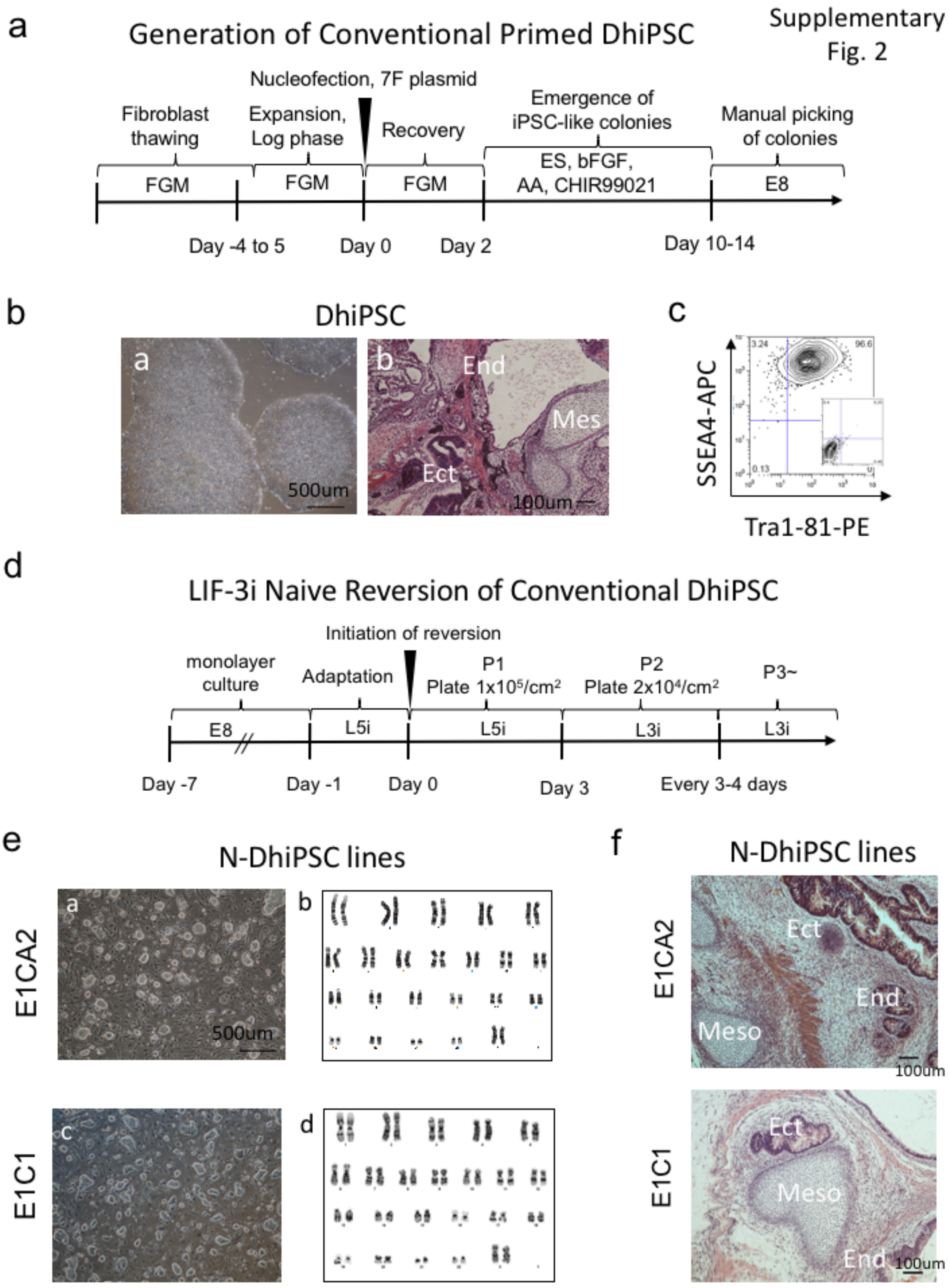

Supplementary Fig. 2
a
Generation of Conventional Primed DhiPSC
Nucleofection, 7F plasmid
Fibroblast thawing
Expansion, Log phase
Recovery
Emergence of iPSC-like colonies
Manual picking of colonies
FGM
FGM
FGM
ES, bFGF, AA, CHIR99021
E8
Day -4 to 5
Day 0
Day 2
Day 10-14
b
DhiPSC
a
500um
b
End
Mes
Ect
100um
c
SSEA4-APC
Tra1-81-PE
3.24
96.6
0.13
d
LIF-3i Naive Reversion of Conventional DhiPSC
Initiation of reversion
monolayer culture
Adaptation
P1 Plate 1x10⁵/cm²
P2 Plate 2x10⁴/cm²
P3~
E8
L5i
L5i
L3i
L3i
Day -7
Day -1
Day 0
Day 3
Every 3-4 days
e
N-DhiPSC lines
E1CA2
E1C1
a
500um
b
c
d
f
N-DhiPSC lines
E1CA2
Ect
End
Meso
100um
E1C1
Ect
Meso
End
100um

Supplementary Figure 3. **APEL vascular differentiation system.** (**a**) Kinetics patterns of surface protein expressions by flow cytometry during APEL vascular differentiation for pluripotency markers (SSEA4, TRA-1-81) and vascular markers (CD31, CD146, CD34, CD144) from isogenic primed (blue) and naïve (red) DhiPSC lines. Shown are mean results with SEM of two independent differentiation experiments of N-DhiPSC and their isogenic primed DhiPSC counterparts (lines E1C1, E1CA1; *n*=2). (**b**) Shown are indicated growth factors and inhibitor molecules, as described in Methods. Differentiation cultures were enriched for CD31-expressing VP using magnetic-activated cell sorting (MACS); $CD31^+$ VP co-express CD146 post-MACS enrichment. These $CD31^+CD146^+$ VP populations were further expanded for several passages in EGM2 medium prior to *in vitro* characterization or injection into I/R-injured murine NOG eyes.

a

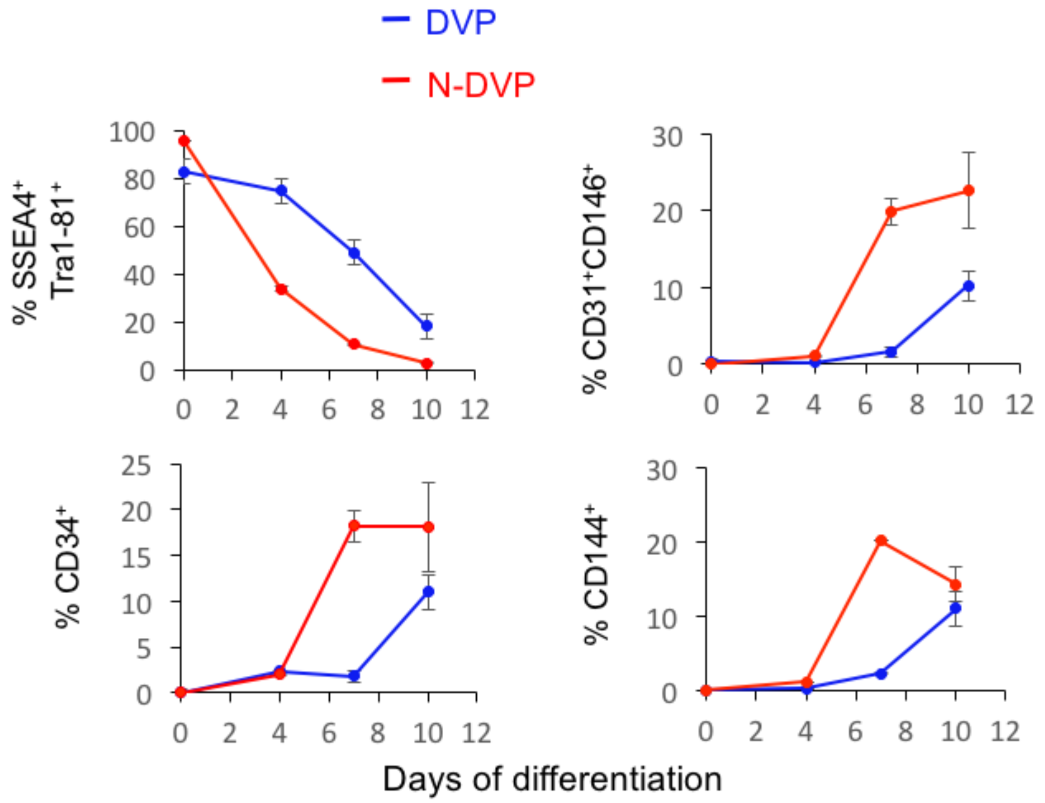

DVP
N-DVP
% SSEA4+ Tra1-81+
% CD31+CD146+
% CD34+
% CD144+
Days of differentiation

b

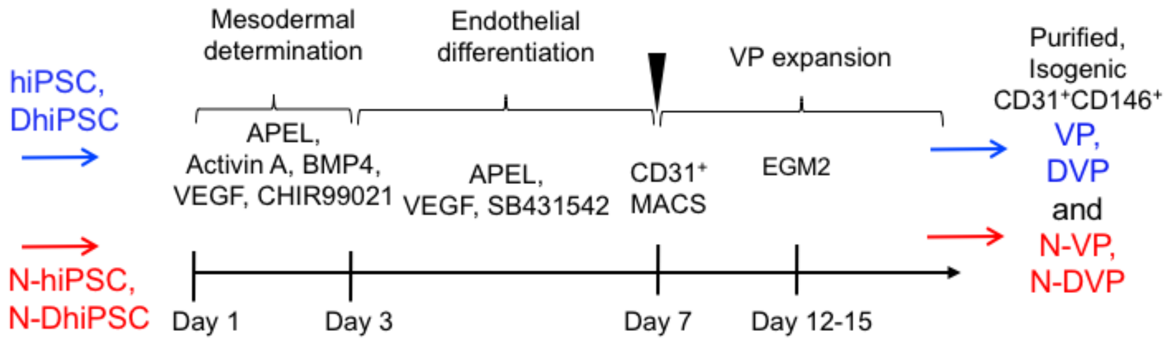

hiPSC,
DhiPSC
N-hiPSC,
N-DhiPSC
Mesodermal determination
Endothelial differentiation
VP expansion
APEL,
Activin A, BMP4,
VEGF, CHIR99021
APEL,
VEGF, SB431542
CD31+
MACS
EGM2
Day 1
Day 3
Day 7
Day 12-15
Purified,
Isogenic
CD31+CD146+
VP,
DVP
and
N-VP,
N-DVP

Supplementary Figure 4. **APEL vascular differentiation of primed and naïve DhiPSC and FACS gating strategy for $CD31^{+}CD146^{+}$ VP purifications**. (**a**) Both primed (E8) and naïve (LIF-3i) DhiPSC (line E1C1) differentiated efficiently using the APEL monolayer vascular differentiation system. Shown are morphologies of APEL-differentiated DVP cells before and after CD31-sorting and re-plating in EGM2. (**b**) Representative flow cytometry expressions of CD31 and CD146 prior to and post sorting of APEL differentiation cultures with CD31 magnetic bead-tagged antibody (MACS: magnetic activated cell sorting). (**c**) Flow cytometry analysis post CD31-sort (prior to EGM2 expansion) vascular lineage surface marker analysis of primed DVP (left panel) vs N-DVP (right panel) demonstrating no significant differences in vascular marker expressions post CD31-sorting from APEL cultures. (**d**) $CD31^{+}CD146^{+}$ N-VP and N-DVP were generated from normal non-diabetic naïve N-CBiPSC and naïve N-DhiPSC, respectively, and analyzed post-CD31 sorting for surface expressions of vascular markers (*e.g*., CD31, CD146, CD144, CXCR4, CD90, and CD105); which demonstrated no significant differences.

Supplementary Fig. 4

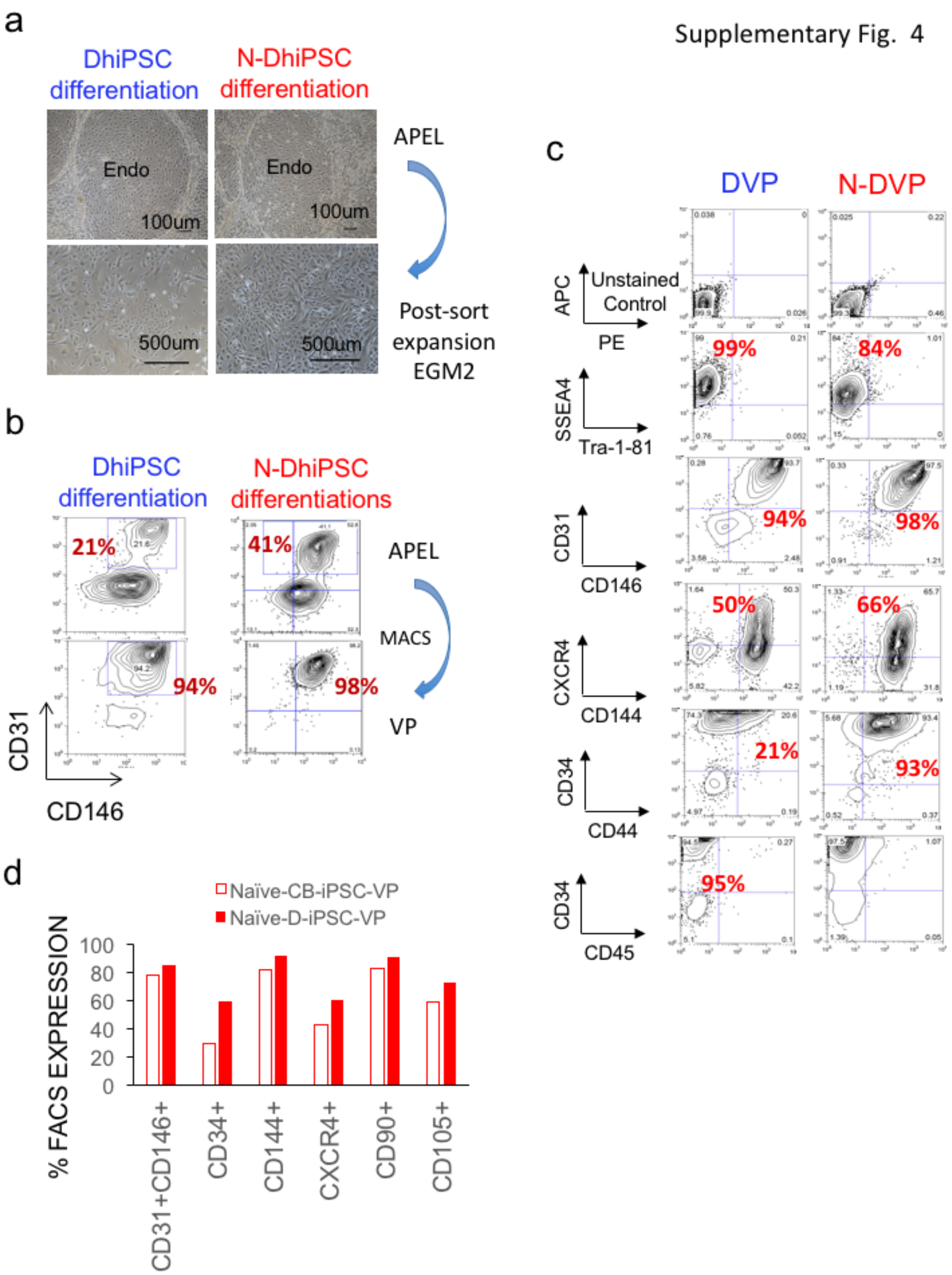

Supplementary Figure 5. ***In vitro* vascular function of DVP vs. N-DVP.** (**a**) Representative images of β-galactosidase senescence assays at passage 4 post re-plating of primed VP vs. N-VP from a normal fibroblast-hiPSC (C1.2), and primed DVP vs N-DVP from a diabetic hiPSC line (E1C1). (**b**) Matrigel vascular tube formation assay demonstrated that N-DVP formed longer and more mature types of tubes than primed DVP. (**c**) (Left panel) representative flow cytometry analysis of EdU assays at passage 1 and passage 3 post re-plating of primed DVP vs. N-DVP from a diabetic hiPSC line (E1C1) (see **Figure 4e**). (Right panel) EdU proliferation assays of purified DVP after 1-5 passages in EGM2 post-$CD31^+$ purification. DhiPSC line E1CA2; *n*=2 independent experiments.

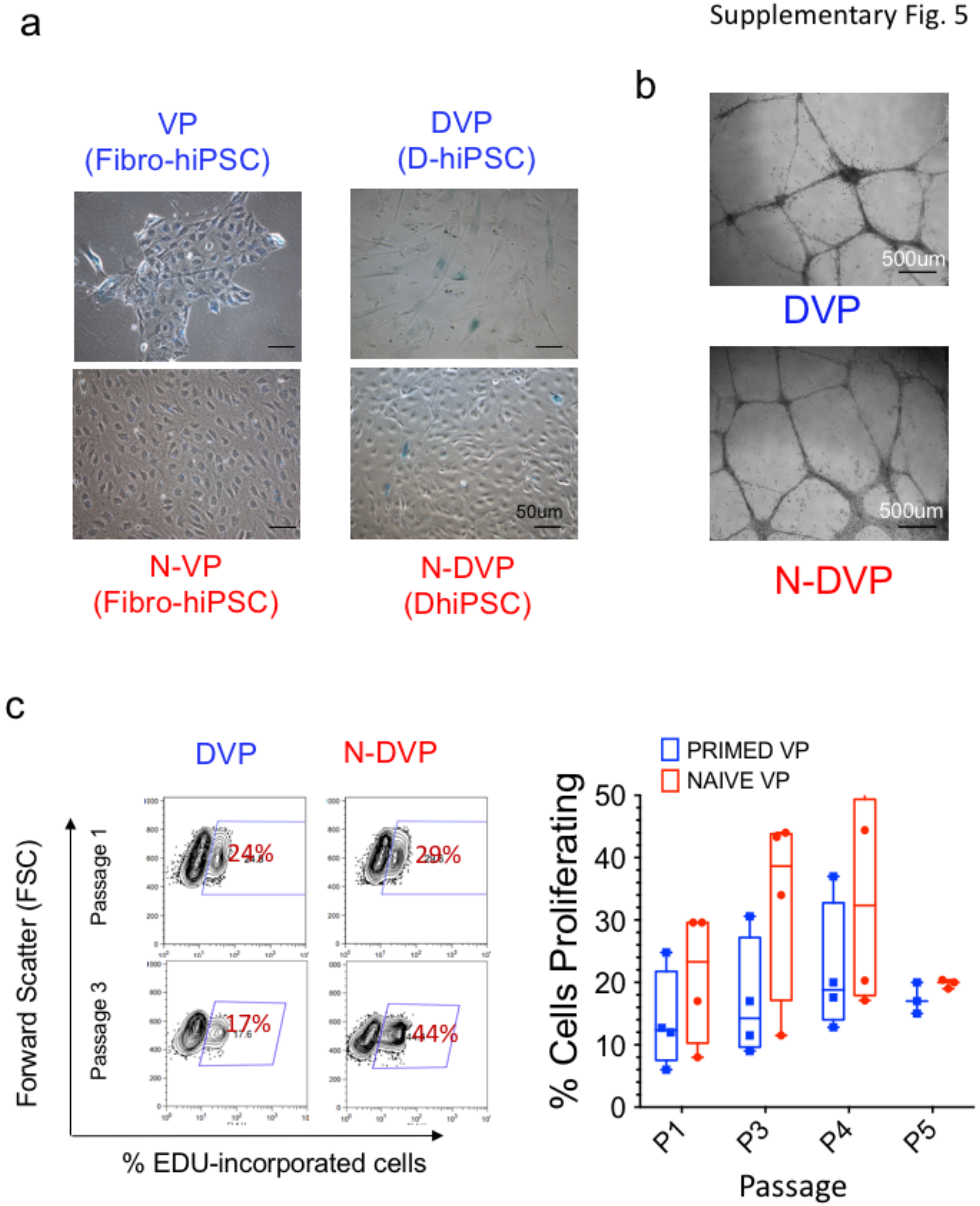
a
VP
(Fibro-hiPSC)
DVP
(D-hiPSC)
50um
N-VP
(Fibro-hiPSC)
N-DVP
(DhiPSC)
b
500um
DVP
500um
N-DVP
c
DVP
N-DVP
Forward Scatter (FSC)
Passage 1
Passage 3
24%
29%
17%
44%
% EDU-incorporated cells
PRIMED VP
NAIVE VP
% Cells Proliferating
50
40
30
20
10
0
P1
P3
P4
P5
Passage

Supplementary Figure 6. **Western blot analysis of DNA damage response (DDR) proteins following treatment of primed VP and N-VP with NCS.** Purified and re-plated VP vs N-VP differentiated from isogenic primed normal fibroblast-hiPSC (C2); with (+) and without (-) NCS treatment prior cell lysate collection. P-DNA-PK: phosphorylated DNA-PK; P-H2AX: phosphorylated H2AX; NCS: neocarzinostatin.

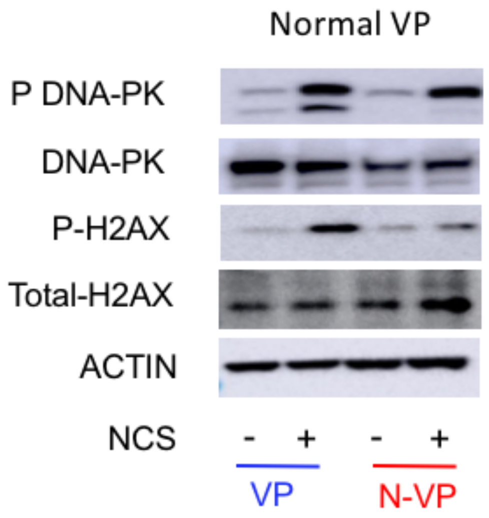
Normal VP
P DNA-PK
DNA-PK
P-H2AX
Total-H2AX
ACTIN
NCS
- +
- +
VP
N-VP

Supplementary Figure 7. **Human VP cell therapy of murine ischemic retinopathy following I/R injury**. Representative high magnification images from whole mount retinae demonstrating more abundant survival of HNA^+ N-DVP cells in the superficial vascular layers of the retina relative to primed DVP at (**a**) 7 days and (**b**) three weeks following parallel intra-vitreal injections of 50,000 DVP or N-DVP cells per eye. (**b**) Retinal vascular regions at 3 weeks demonstrated that HNA^+ human DVP cells were located either abundantly in suspension within vitreous or had migrated and engrafted into vascular abluminal regions of murine ischemic vessels (arrow heads). Scale bars = 50 μm. Whole mount retinae were also stained with anti- murine CD31 (mCD31) or anti-murine collagen IV (mCoIV) antibodies for maximum-intensity projection imaging. (**c,d**) Human vascular engraftment of murine retinae at 2 weeks post DVP injection. (**c**) Whole mount retinal staining of mouse CD31 (mCD31) and co-staining with murine collagen type-IV (mCol-IV) at 2 weeks following I/R injury and naïve (N-DVP) injections; (**d**) co-staining with human CD34 (hCD34)-specific and murine collagen type-IV (mCol-IV) antibodies of similar whole mount retinal samples demonstrated that a subset of these $mCD31^+mCol^+$ murine vessels had engrafted $CD34^+$ vascular cells.

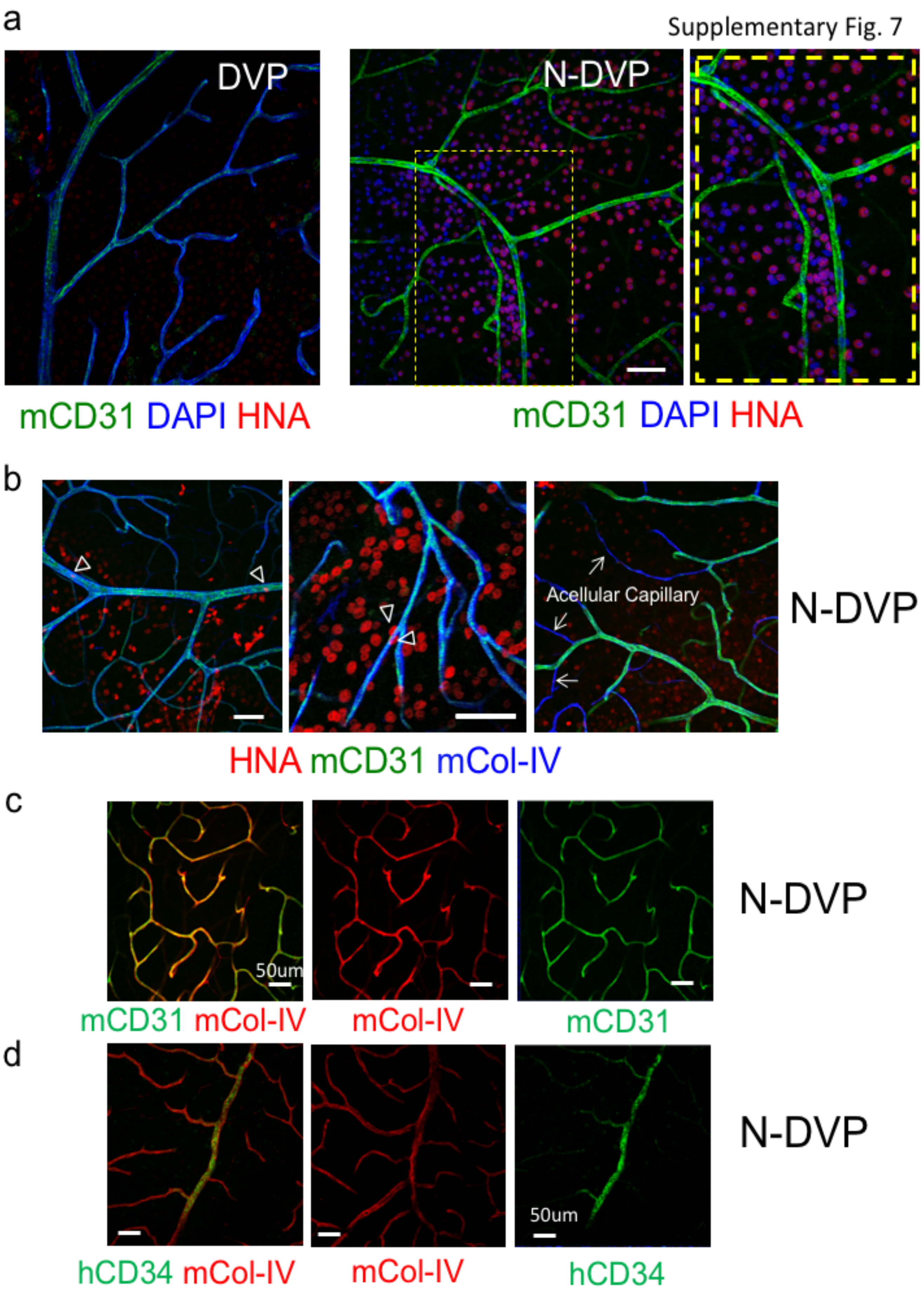
a
DVP
N-DVP
mCD31 DAPI HNA
mCD31 DAPI HNA
b
Acellular Capillary
N-DVP
HNA mCD31 mCol-IV
c
50um
N-DVP
mCD31 mCol-IV
mCol-IV
mCD31
d
50um
N-DVP
hCD34 mCol-IV
mCol-IV
hCD34

Supplementary Figure 8. **Method of quantitating human DVP cell engraftment in the vasculature of the neural layers of ischemia-injured mouse retinae**. (**a**) Tile-scanned image of cryo-sectioned retina. Multiple images were taken in 20x magnification from each cryo-sections as well as spaced (serial sectioned) retina sections for quantification studies. Hatched areas demonstrate representative example of neural retinal regions evaluated for human vascularization of murine blood vessels. (**b**) Method of regional separation of neural retinal layers employed for quantification of human $CD34^+$ cells (hCD34) for data presented in **Figure 7b** and **7d** via automated counts using FiJi distribution of ImageJ software, as described in Methods.

Supplementary Fig. 8

a

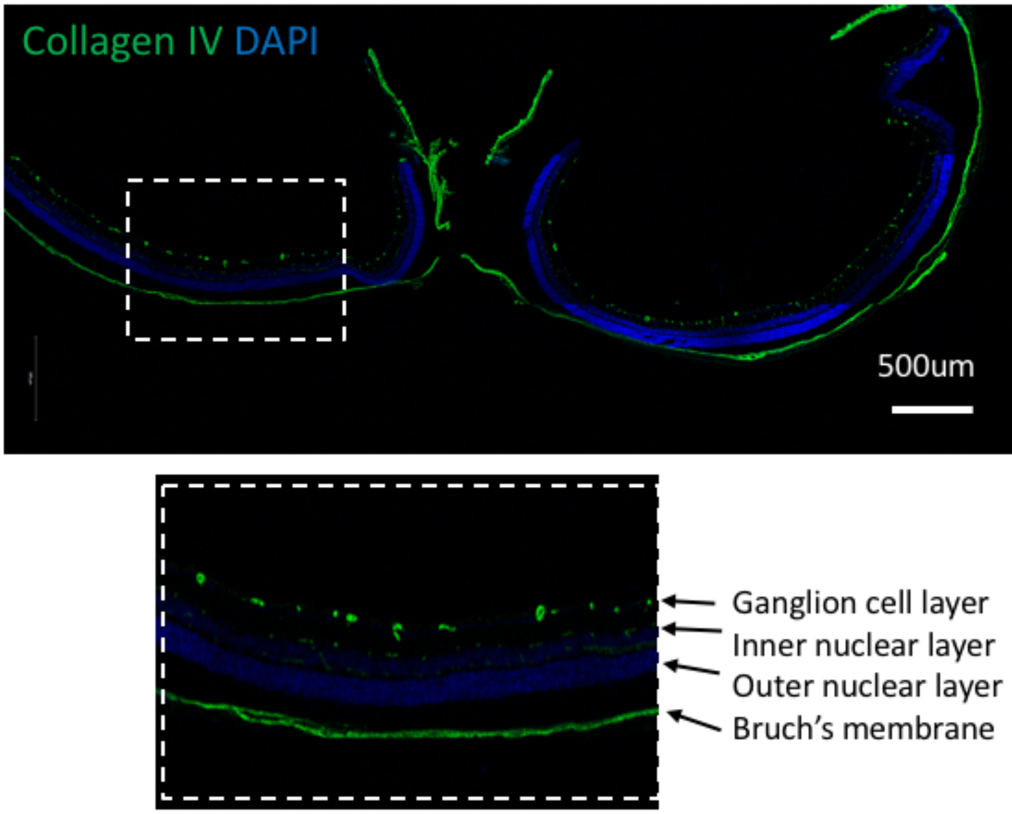

b

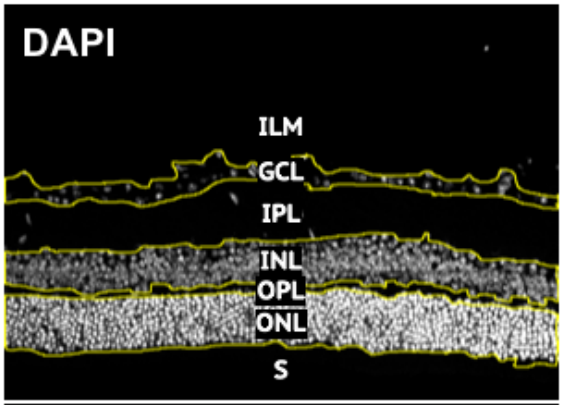

inner limiting membrane	ILM
ganglion cell layer	GCL
inner plexiform layer	IPL
inner nuclear layer	INL
outer plexiform layer	OPL
outer nuclear layer	ONL
segments	S

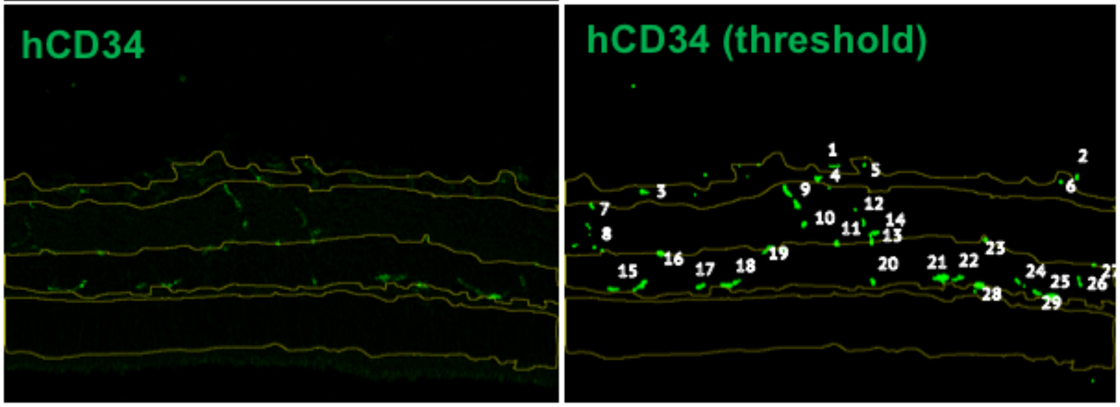

Supplementary Figure 9. **Lineage-primed gene expression and epigenetic configurations of PRC2-regulated bivalent promoters in primed vs naïve DhiPSC**. (**a**) Crossplot of mean genome-wide gene expression of PRC2 module gene targets (**Supplementary Table 3**) *vs.* CpG methylation of non-diabetic N-hiPSC *vs.* their isogenic primed isogenic hiPSC counterparts (*n*=6 normal hiPSC lines; **Table S1**). Plotted are the differentially methylated region (DMR) CpG methylation beta values of PRC2 module promoter regions in LIF-3i-reverted hiPSC minus their isogenic primed hPSC counterparts (y-axis, p ≤0.05) *vs*. their corresponding differential gene expressions for the same genes (red, x-axis, log_2 fold changes (FC); p ≤ 0.05, FC +/- ≥1.5.). **(b)** Curated GSEA pathways for gene targets of the PRC2 module over-represented (FDR<0.01; p<0.001) in LIF-3i-reverted fibroblast-hiPSC *vs*. their isogenic primed counterparts (*n*=4; normal hiPSC lines – **Supplementary Table 1**). (**c**) Comparison of differentially expressed (p ≤ 0.05, fold change (FC) +/- ≥1.5) lineage-primed transcriptional targets of the PRC2 module (**Supplementary Table 3**) in naïve vs primed hiPSC (*n*=6 independent lines). FC: normalized ratios of naïve/primed expression microarray signal intensities show broadly decreased expressions of PRC2 targets in N-hiPSC lines. Ratios are of LIF-3i-reverted *vs.* primed hPSC samples. (**d**) ChiP-qPCR for H3K27me3 and H3K4me3 histone marks at key multi-lineage developmental promoters in primed vs N-DhiPSC line E1C1. Data is presented as percent input materials between naïve and primed D-hiPSC line E1C1. Standard error of the mean (SEM) bar represents replicates. ChiP-PCR primer sequences and sources are detailed in **Supplementary Table 3**.

Supplementary Fig. 9

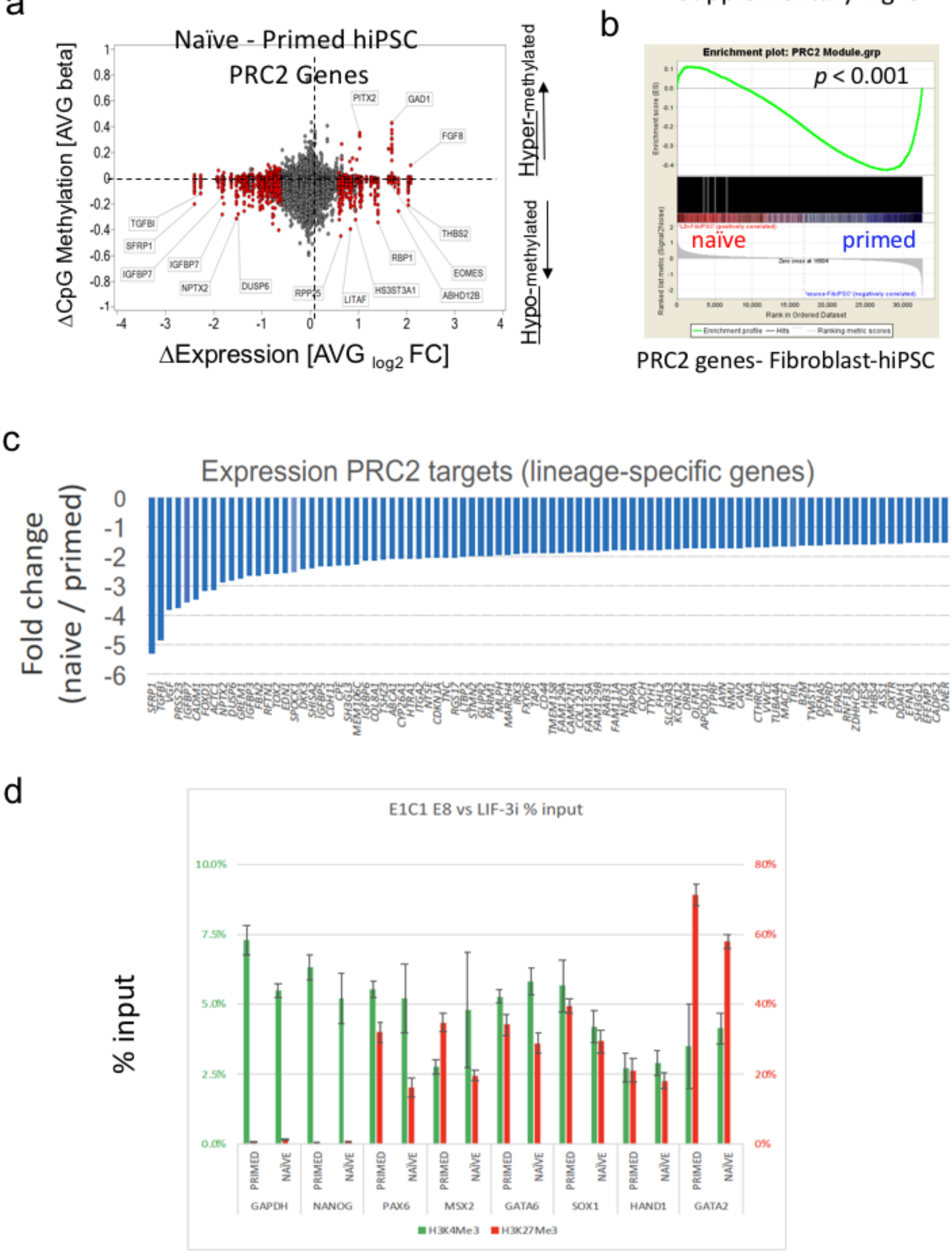

Supplement: Supplementary file 1 — Supplementary Information [file 41467_2020_14764_MOESM1_ESM.pdf]
