# Supplementary material for: Vascular progenitors generated from tankyrase inhibitor-regulated naïve diabetic human iPSC potentiate efficient revascularization of ischemic retina
Source: Nat Commun. 2020 Mar 5;11:1195. doi: 10.1038/s41467-020-14764-5 (PMC7058090; doi:10.1038/s41467-020-14764-5)
Supplement: Supplementary file 3 — Description of Additional Supplementary Files [file 41467_2020_14764_MOESM3_ESM.docx]

**Description of Additional Supplementary Files**

File Name: Supplementary Data 1

Description: Human iPSC lines and naïve reversion methods

File Name: Supplementary Data 2

Description: Human iPSC cell line karyotype summary

File Name: Supplementary Data 3

Description: Antibodies, PCR primers, and lineage-specific gene expression

(PRC2 module)

File Name: Supplementary Data 4

Description: RNA-Seq: differential gene expression of primed vs. naïve normal

and diabetic VP (top 500 genes)

File Name: Supplementary Data 5

Description: RNA-Seq: differential gene expression of normal and diabetic naïve hPSC vs. normal and diabetic primed hPSC
