# Supplementary material for: Vascular progenitors generated from tankyrase inhibitor-regulated naïve diabetic human iPSC potentiate efficient revascularization of ischemic retina
Source: Nat Commun. 2020 Mar 5;11:1195. doi: 10.1038/s41467-020-14764-5 (PMC7058090; doi:10.1038/s41467-020-14764-5)
Supplement: Supplementary file 9 — Reporting Summary [file 41467_2020_14764_MOESM9_ESM.pdf]

nature research

Corresponding author(s): Elias T. Zambidis MD/PhD

Last updated by author(s): December 9, 2019

Reporting Summary

Nature Research wishes to improve the reproducibility of the work that we publish. This form provides structure for consistency and transparency in reporting. For further information on Nature Research policies, see Authors & Referees and the Editorial Policy Checklist .

Statistics

For all statistical analyses, confirm that the following items are present in the figure legend, table legend, main text, or Methods section.

n/a	Confirmed	
☐	☒	The exact sample size (*n*) for each experimental group/condition, given as a discrete number and unit of measurement
☐	☒	A statement on whether measurements were taken from distinct samples or whether the same sample was measured repeatedly
☐	☒	The statistical test(s) used AND whether they are one- or two-sided<br>*Only common tests should be described solely by name; describe more complex techniques in the Methods section.*
☐	☒	A description of all covariates tested
☒	☐	A description of any assumptions or corrections, such as tests of normality and adjustment for multiple comparisons
☐	☒	A full description of the statistical parameters including central tendency (e.g. means) or other basic estimates (e.g. regression coefficient) AND variation (e.g. standard deviation) or associated estimates of uncertainty (e.g. confidence intervals)
☐	☒	For null hypothesis testing, the test statistic (e.g. *F*, *t*, *r*) with confidence intervals, effect sizes, degrees of freedom and *P* value noted<br>*Give P values as exact values whenever suitable.*
☒	☐	For Bayesian analysis, information on the choice of priors and Markov chain Monte Carlo settings
☒	☐	For hierarchical and complex designs, identification of the appropriate level for tests and full reporting of outcomes
☒	☐	Estimates of effect sizes (e.g. Cohen's *d*, Pearson's *r*), indicating how they were calculated

Our web collection on statistics for biologists contains articles on many of the points above.

Software and code

Policy information about availability of computer code

Data collection	1. confocal microscopy - ZEN software
Data analysis	1. confocal microscopy - ZEN software 2. densitometry - ImageJ software 3. Bioinformatics - Illumina GenomeStudio, Spotfire, and Partek statistics package; RNA-Seq Bioinformatics: BioConductor, DSeq2

For manuscripts utilizing custom algorithms or software that are central to the research but not yet described in published literature, software must be made available to editors/reviewers. We strongly encourage code deposition in a community repository (e.g. GitHub). See the Nature Research guidelines for submitting code & software for further information.

Data

Policy information about availability of data

All manuscripts must include a data availability statement. This statement should provide the following information, where applicable:

- Accession codes, unique identifiers, or web links for publicly available datasets
- A list of figures that have associated raw data
- A description of any restrictions on data availability

All raw data is available as a "Source Data' file. Gene expression and CpG methylation data is publically available in Omnibus under accession numbers GSE65211 and GSE65214, respectively. The NIH Gene Expression Omnibus has issued the accession number GSE141639 for RNA-Seq data in this manuscript.

Field-specific reporting

Please select the one below that is the best fit for your research. If you are not sure, read the appropriate sections before making your selection.

Life sciences study design

All studies must disclose on these points even when the disclosure is negative.

Sample size	sample sizes for each individual experiment is detailed in every figure legend.
Data exclusions	N/A
Replication	Replication of each sample sizes for each individual experiment is detailed in every figure legend, as well as described in Methods section for each individual technique .
Randomization	Randomization was not applicable in the experimental design of these studies
Blinding	Blinding was not applicable in the experimental design of these studies

Reporting for specific materials, systems and methods

We require information from authors about some types of materials, experimental systems and methods used in many studies. Here, indicate whether each material, system or method listed is relevant to your study. If you are not sure if a list item applies to your research, read the appropriate section before selecting a response.

Materials & experimental systems

n/a	Involved in the study	
☐	☒	Antibodies
☐	☒	Eukaryotic cell lines
☒	☐	Palaeontology
☐	☒	Animals and other organisms
☒	☐	Human research participants
☒	☐	Clinical data

Methods

n/a	Involved in the study	
☒	☐	ChIP-seq
☐	☒	Flow cytometry
☒	☐	MRI-based neuroimaging

Antibodies

Antibodies used	All antibodies used in these studies for Western blots, FACS, genomic dot blots, ChiP-PCR, and immunofluorescence, their commercial source, their species, and their method of validation are organized in detail as Supplementary Table S3.
Validation	All antibodies used in these studies for Western blots, FACS, genomic dot blots, ChiP-PCR, and immunofluorescence, their commercial source, their species, and their method of validation are organized in detail as Supplementary Table S3.

Eukaryotic cell lines

Policy information about cell lines

Cell line source(s)	A detailed table of all human pluripotent stem cell lines and their origins used in these studies was composed as Supplementary Table S1
Authentication	The karyotypic and genomic integrity of all human pluripotent stem cell lines used in these studies was compposed as Supplementary Table S2
Mycoplasma contamination	All cell lines used in these studies were confirmed free of mycoplasma contamination
Commonly misidentified lines (See ICLAC register)	None

Animals and other organisms

Policy information about studies involving animals; ARRIVE guidelines recommended for reporting animal research

Laboratory animals	NOD/Shi-scid/IL-2Rγnull) (NOG) mice
Wild animals	N/A
Field-collected samples	N/A

Ethics oversight	Oversight included by IACUC, ISCRO. Details of all bioethics committee oversight of these studies is detailed in Methods Section, 1st paragraph.

Note that full information on the approval of the study protocol must also be provided in the manuscript.

Flow Cytometry

Plots

Confirm that:

- [x] The axis labels state the marker and fluorochrome used (e.g. CD4-FITC).
- [x] The axis scales are clearly visible. Include numbers along axes only for bottom left plot of group (a 'group' is an analysis of identical markers).
- [x] All plots are contour plots with outliers or pseudocolor plots.
- [x] A numerical value for number of cells or percentage (with statistics) is provided.

Methodology

Sample preparation	Details of sample preparation are provided in Methods section, p.22 of the manuscript. Viable cells were analyzed (10,000 events acquired for each sample) using the BD CellQuest Pro analytical software and FACSCalibur™ flow cytometer (BD Biosciences). All data files were analyzed using Flowjo analysis software (Tree Star Inc., Ashland, OR).
Instrument	FACSCalibur (BD Biosciences)
Software	Data acquired with BD CellQuest Pro analytical software, and analyzed with FloJo software (Tree Star, Inc).
Cell population abundance	Details are provided in Methods section.
Gating strategy	Gating strategies and negative controls are provided in multiple sections in Supplementary Data, e.g. in Fig. S2, Fig. S3, and Fig. S4.

- [x] Tick this box to confirm that a figure exemplifying the gating strategy is provided in the Supplementary Information.
