# Supplementary material for: Vascular progenitors generated from tankyrase inhibitor-regulated naïve diabetic human iPSC potentiate efficient revascularization of ischemic retina
Source: Nat Commun. 2020 Mar 5;11:1195. doi: 10.1038/s41467-020-14764-5 (PMC7058090; doi:10.1038/s41467-020-14764-5)

25ug protein/lane

Figure 2b

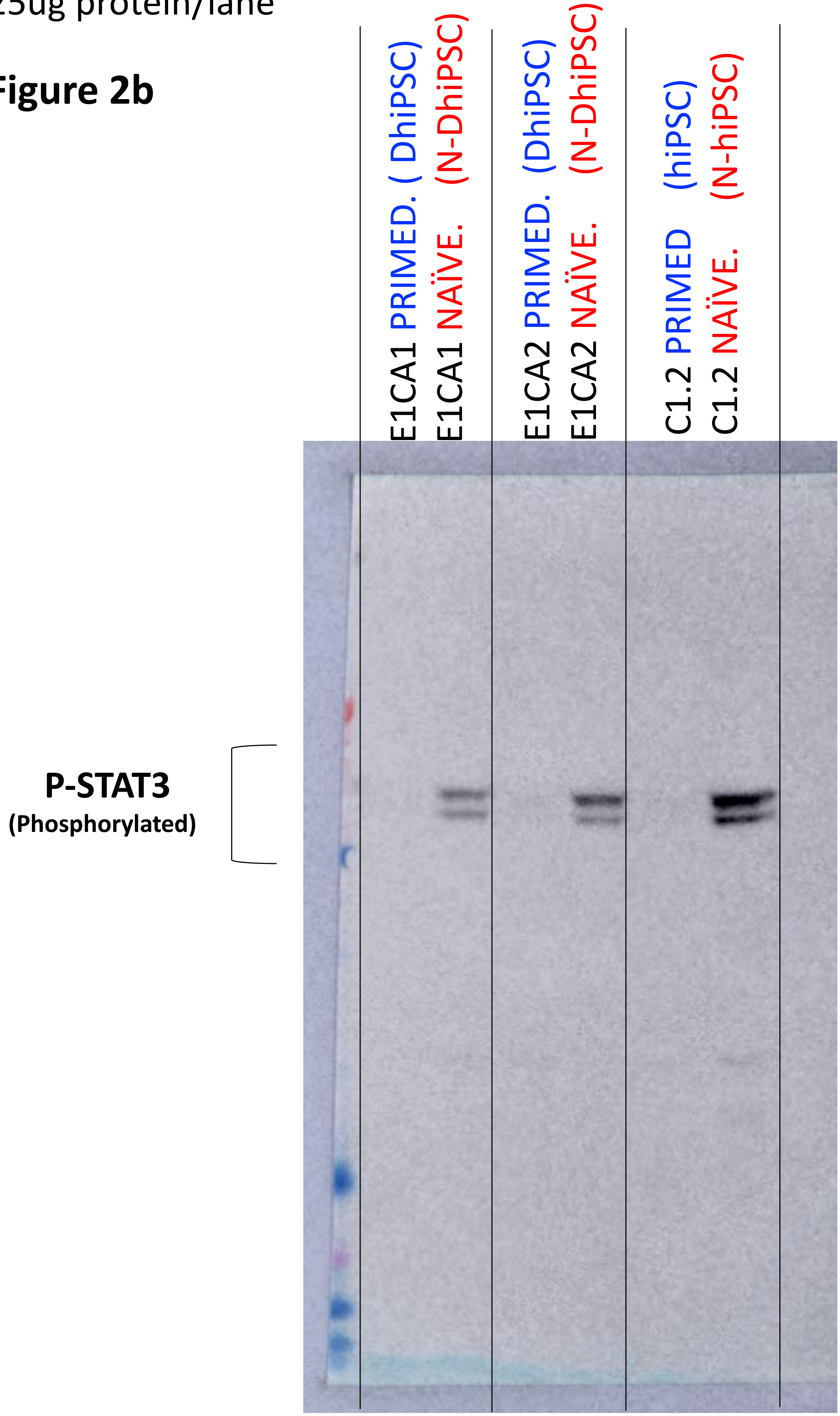

25ug protein/lane

Figure 2b

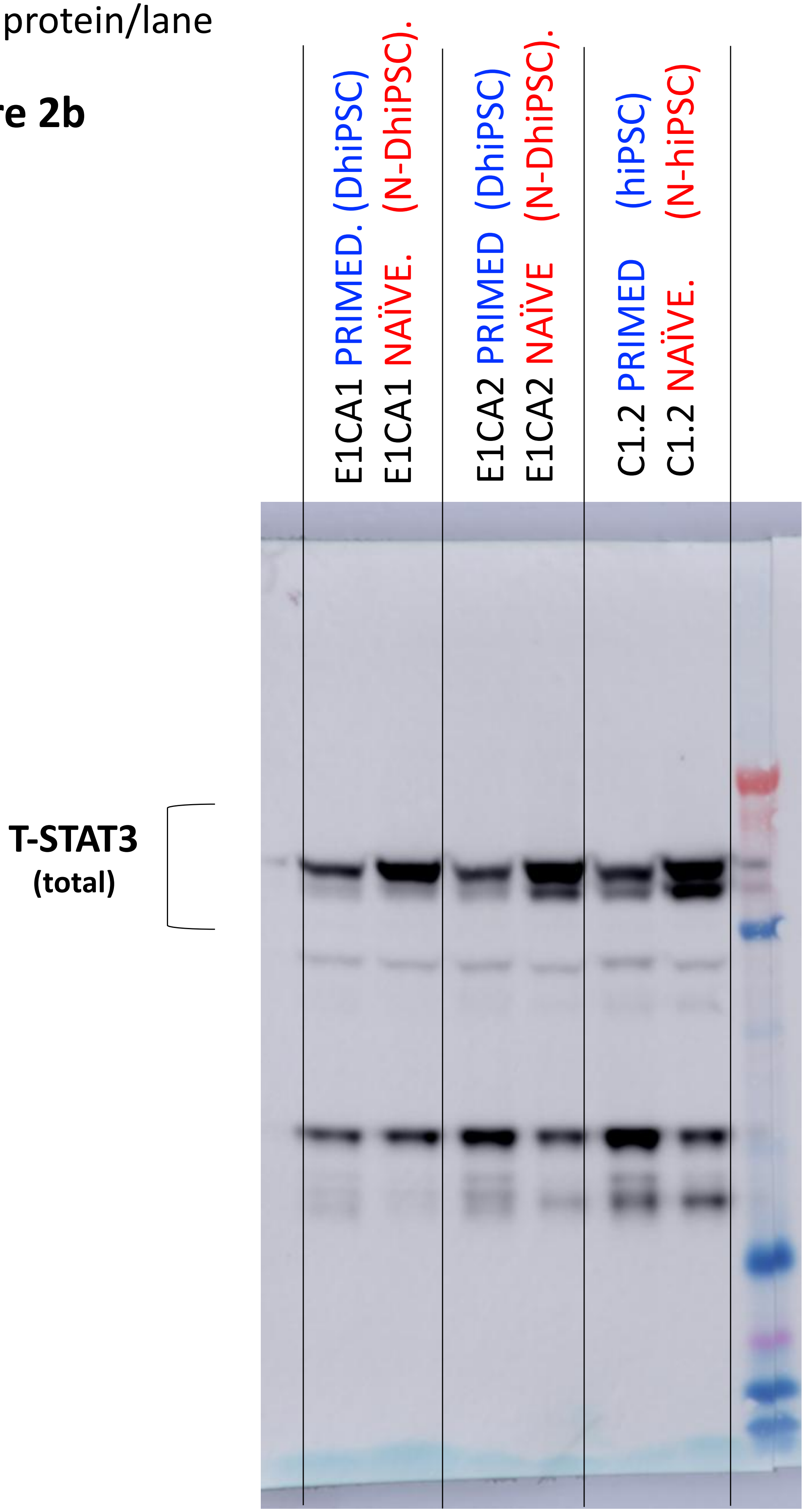

25ug protein/lane

Figure 2b

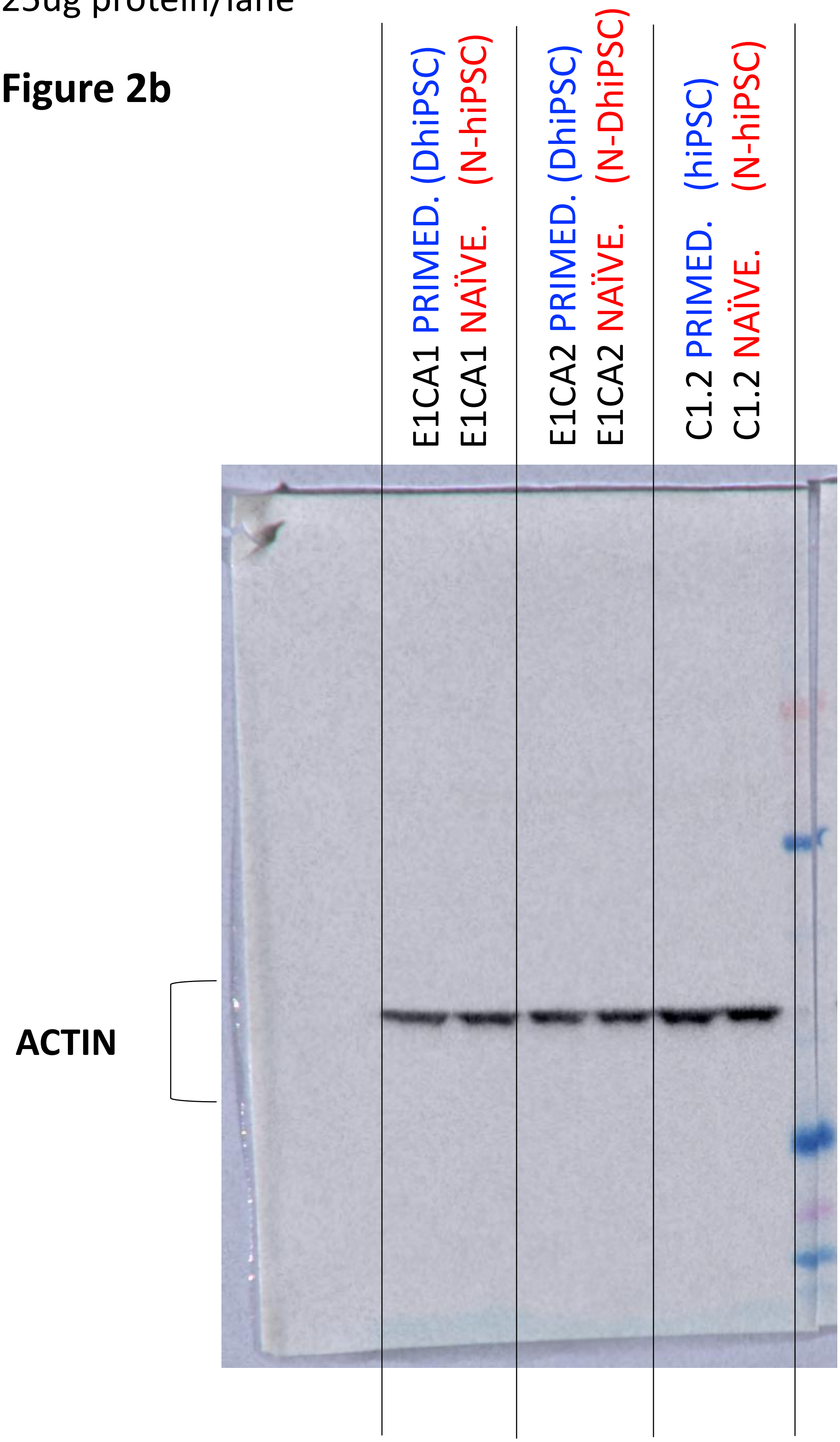

25ug protein/lane

Figure 2b

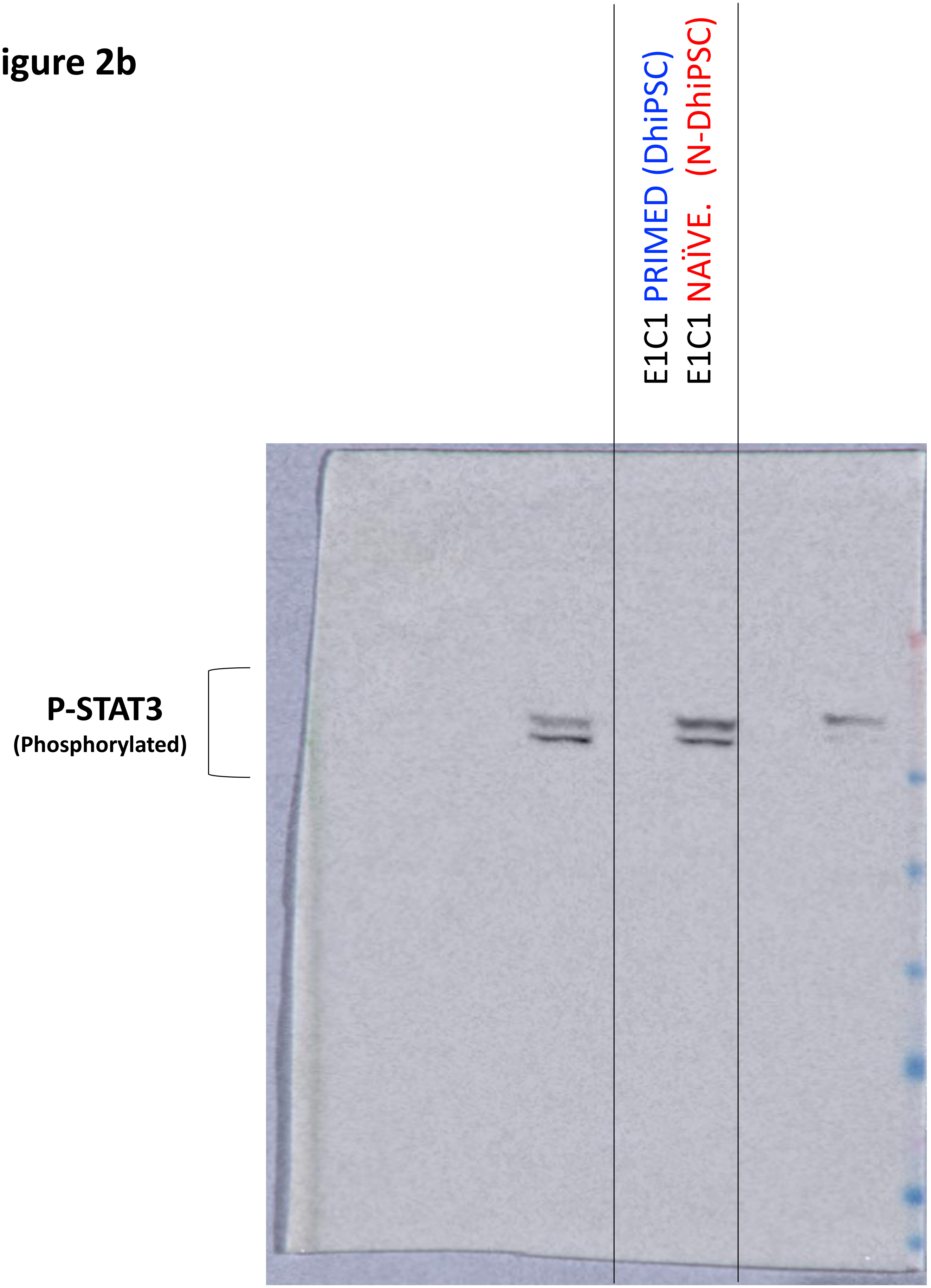

25ug protein/lane

Figure 2b

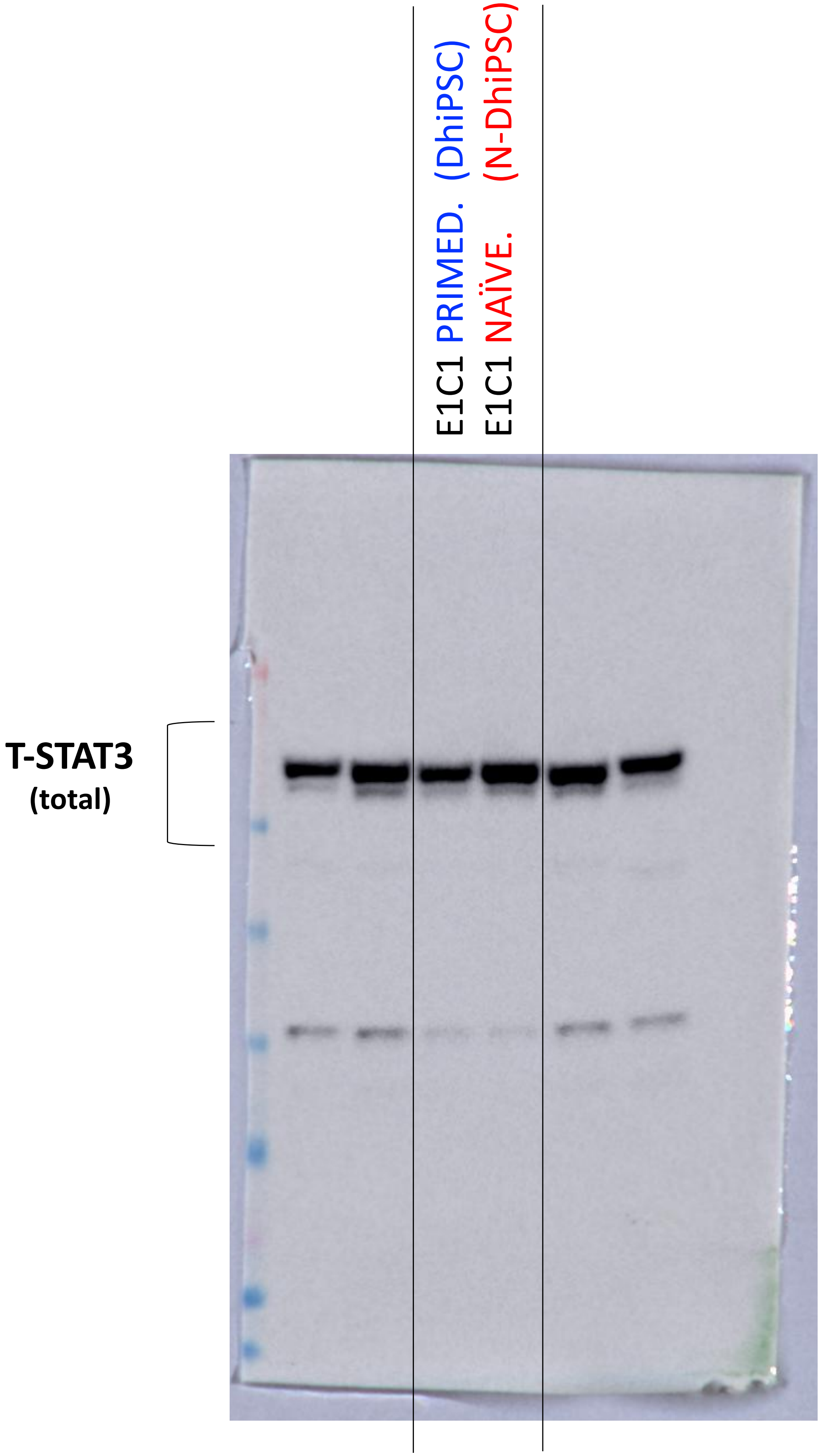

25ug protein/lane

Figure 2b

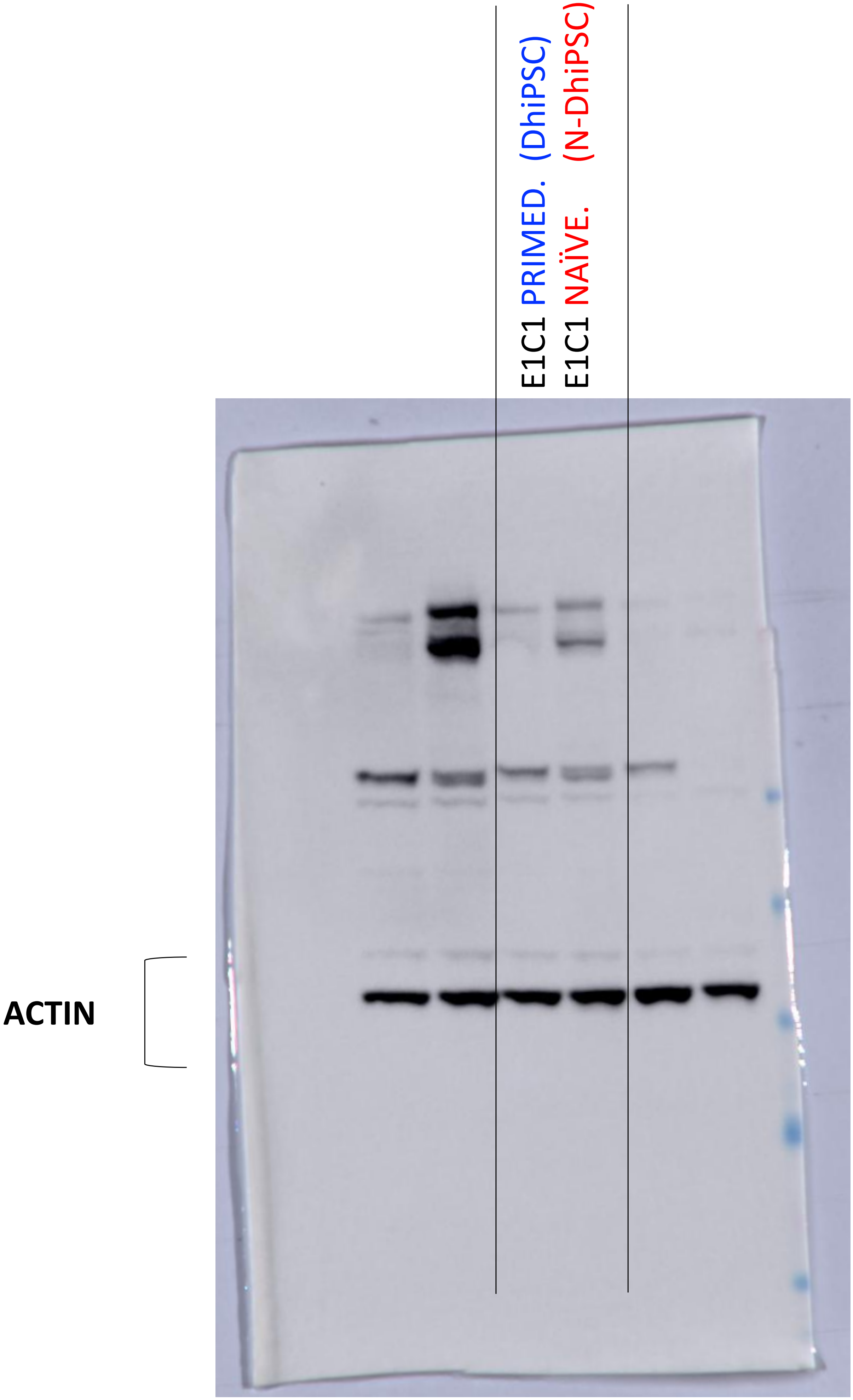

25ug protein/lane

Figure 2b

C2 PRIMED. (hiPSC)

C2 NAÏVE. (DhiPSC)

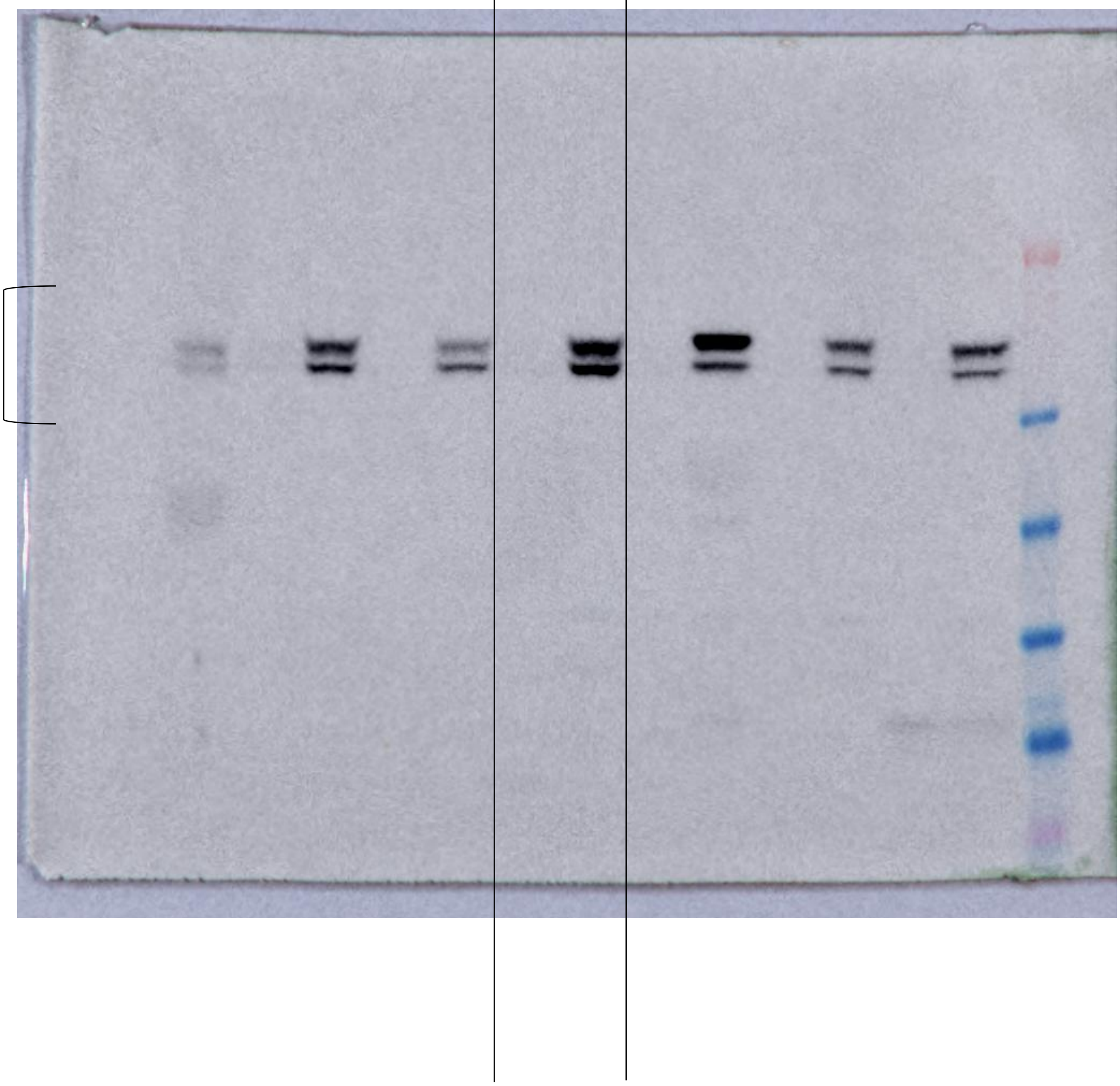

P-STAT3
(Phosphorylated)

25ug protein/lane

Figure 2b

C2 PRIMED. (hiPSC)
C2 NAÏVE. (N-hiPSC)

T-STAT3
(total)

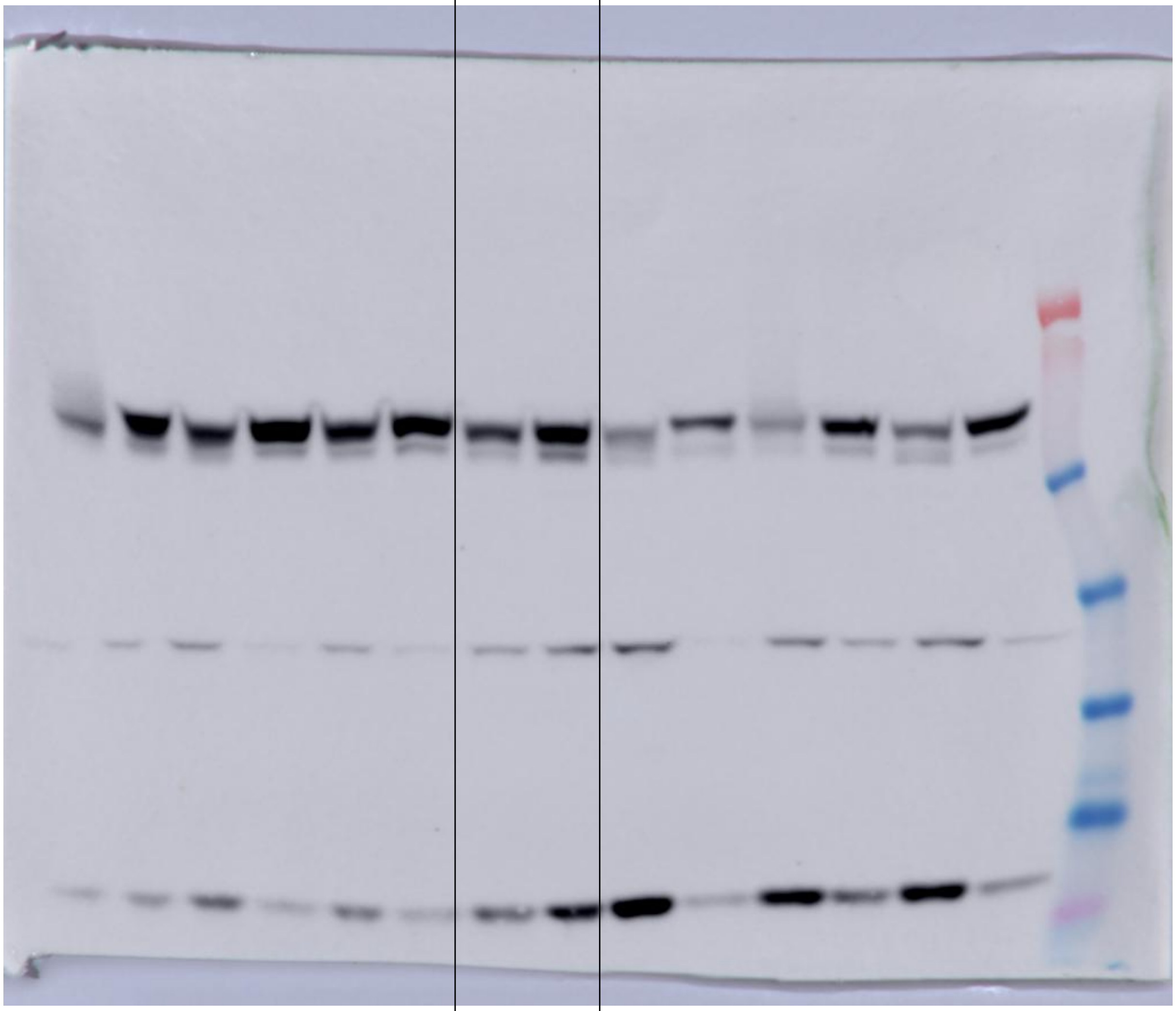

25ug protein/lane

Figure 2b

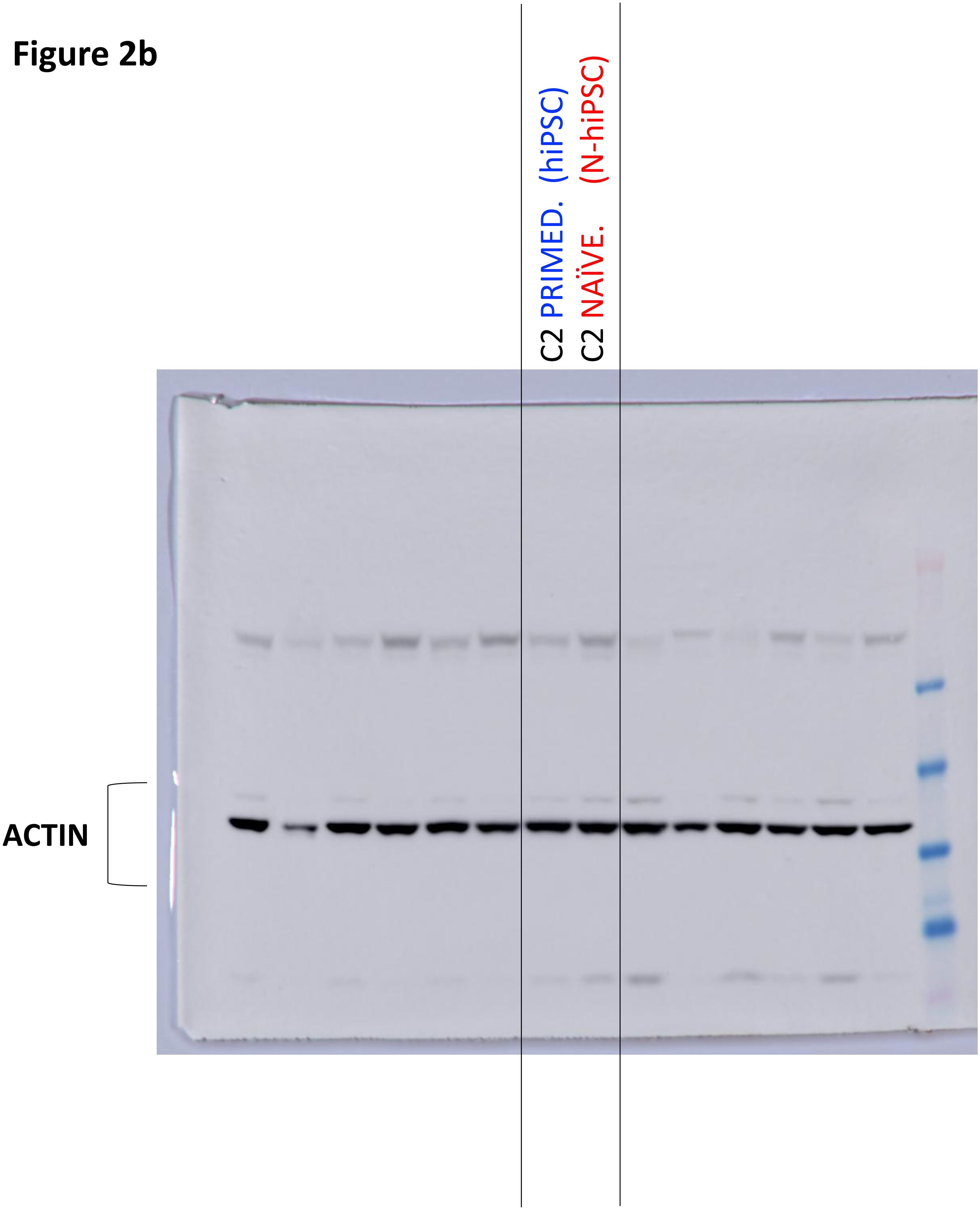

25ug protein/lane

Figure 2c

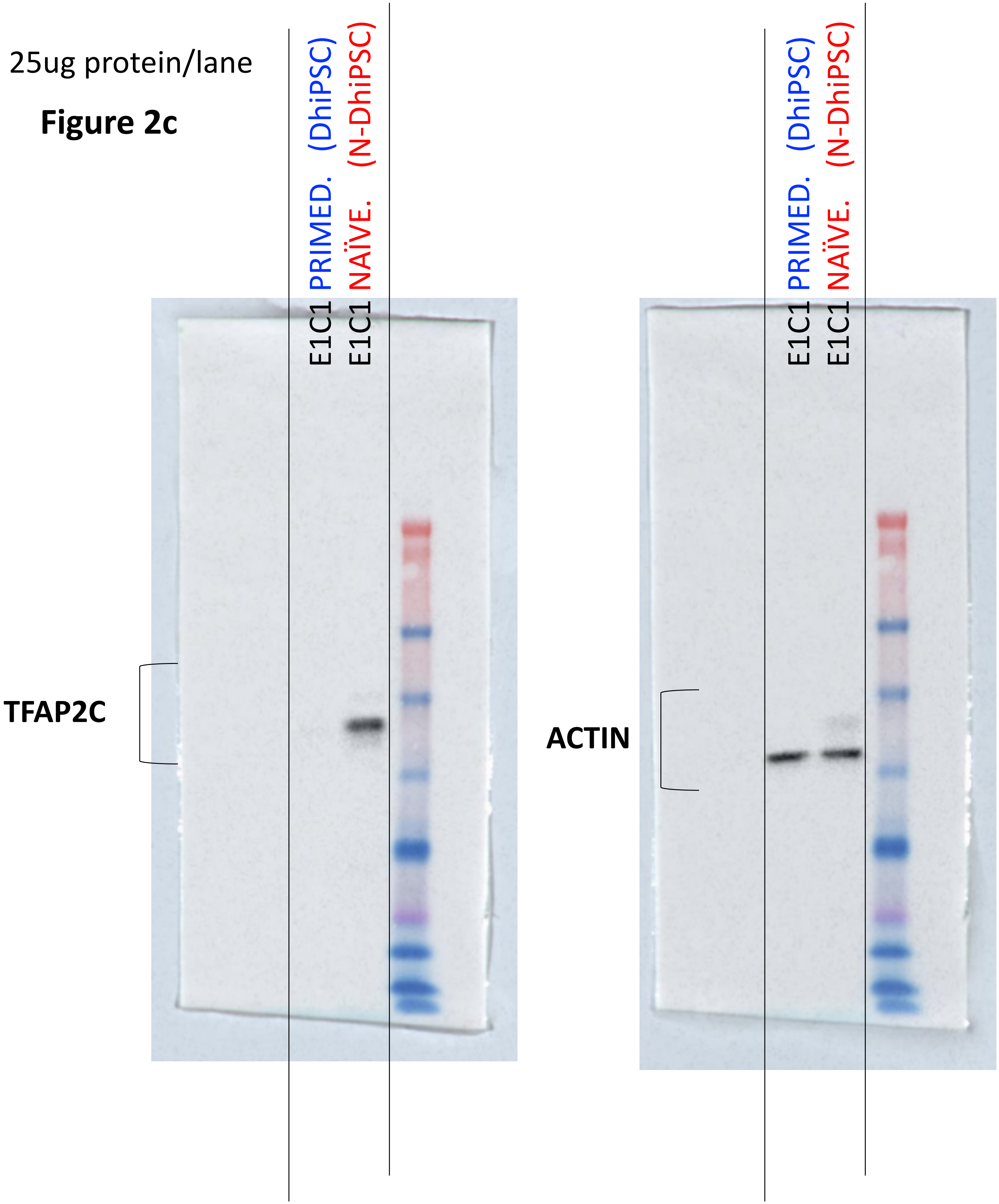

25ug protein/lane

Figure 2e

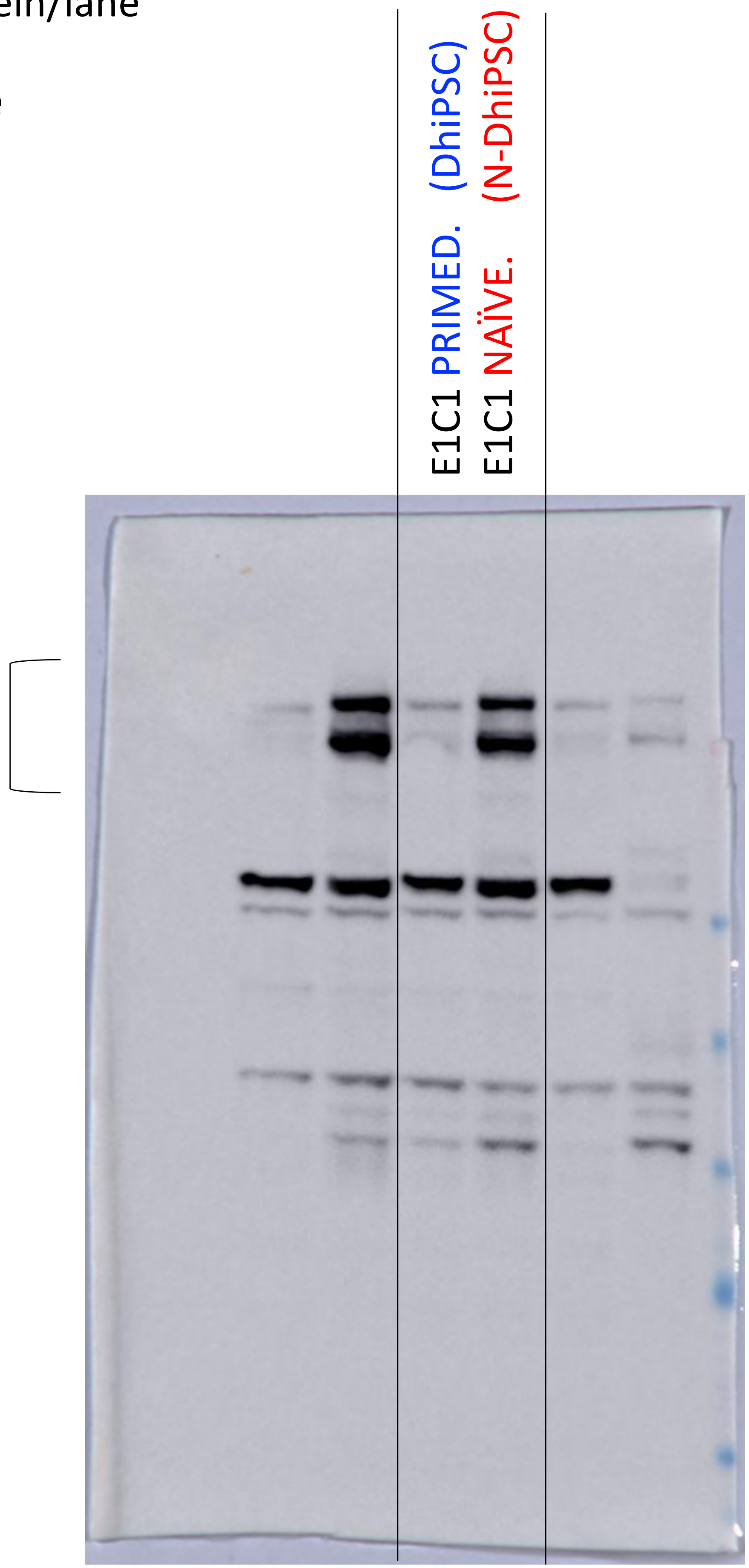

25ug protein/lane

Figure 2e

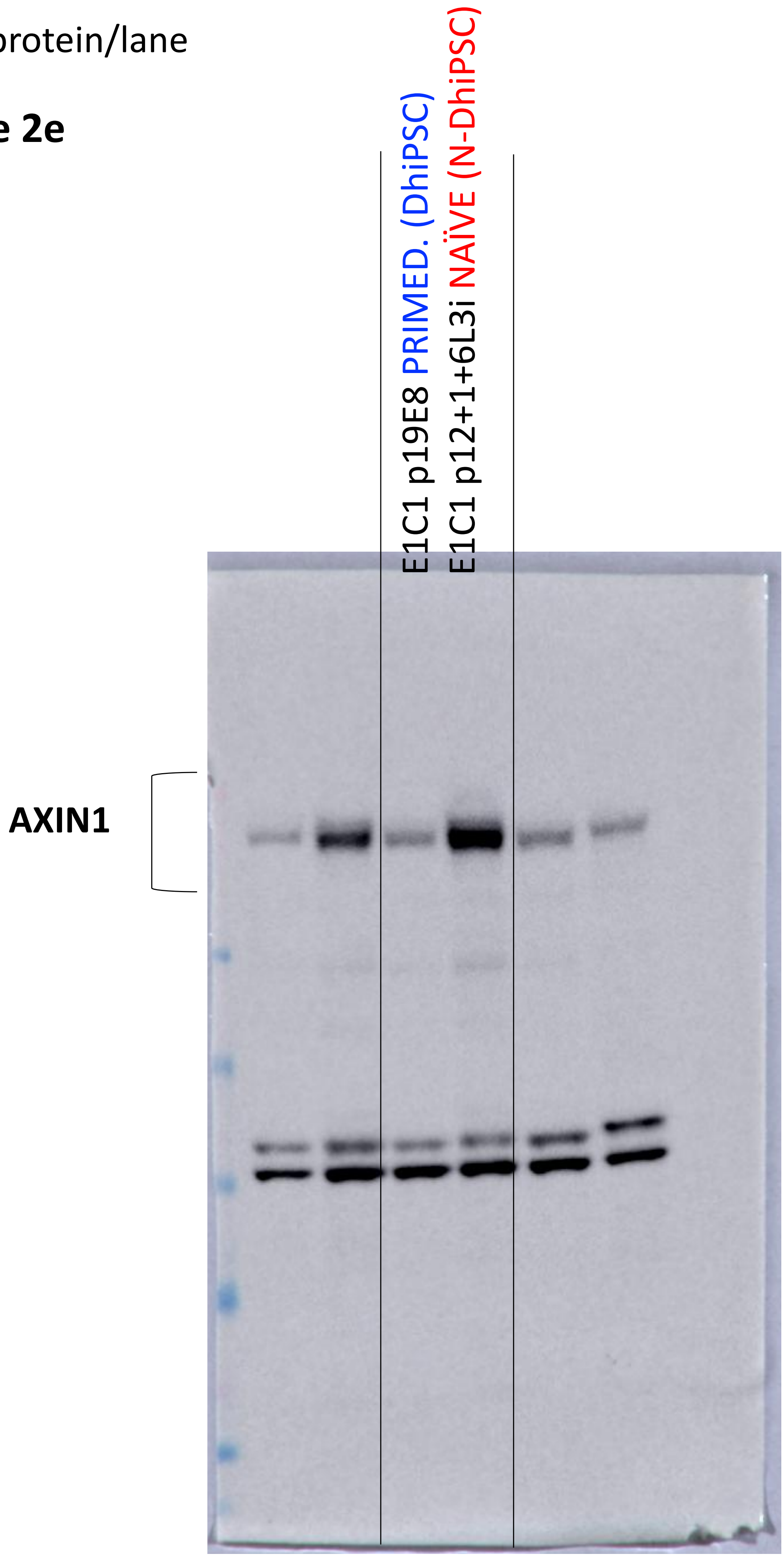

25ug protein/lane

Figure 2e

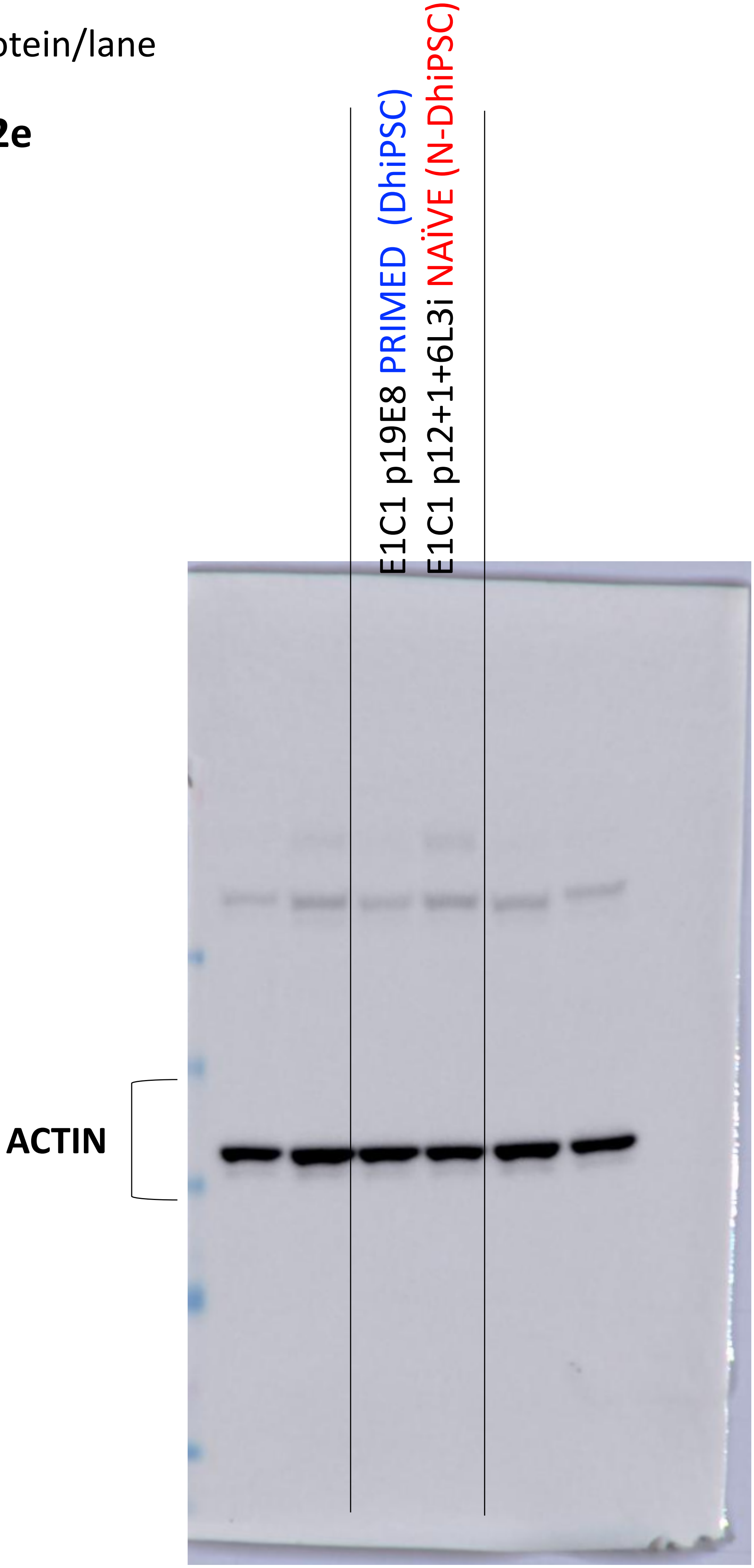

25ug protein/lane

Figure 2e

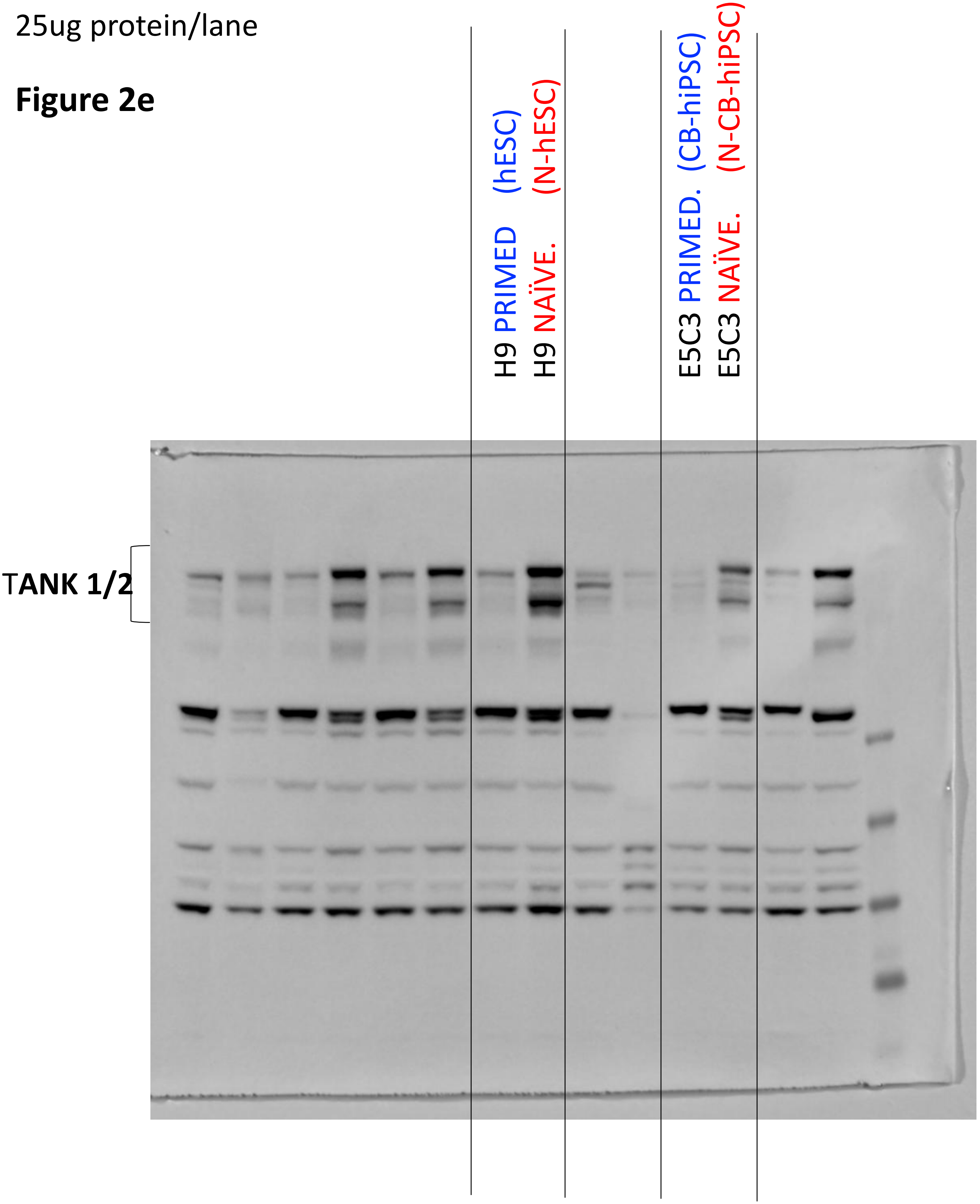

25ug protein/lane

Figure 2e

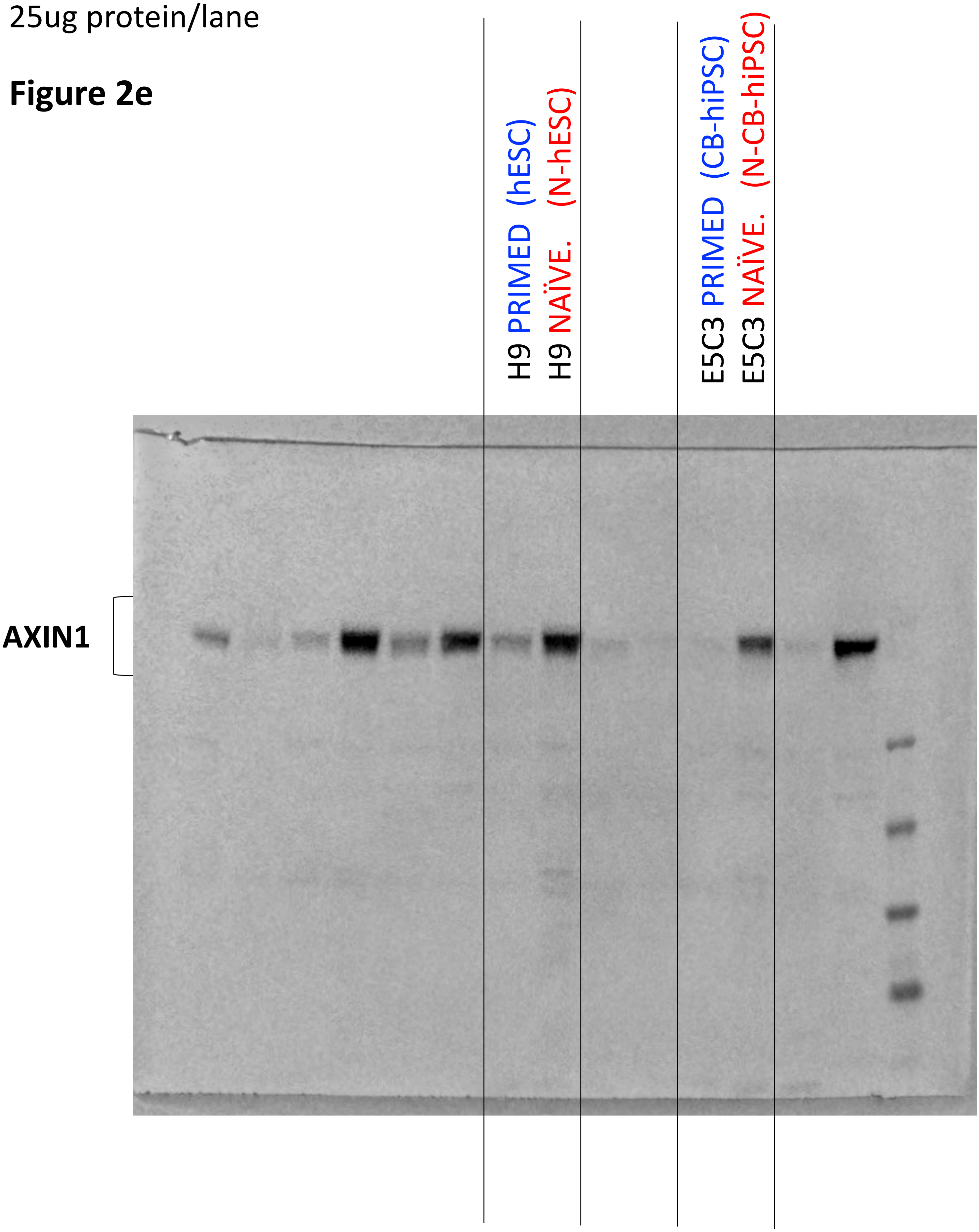

25ug protein/lane

Figure 2e

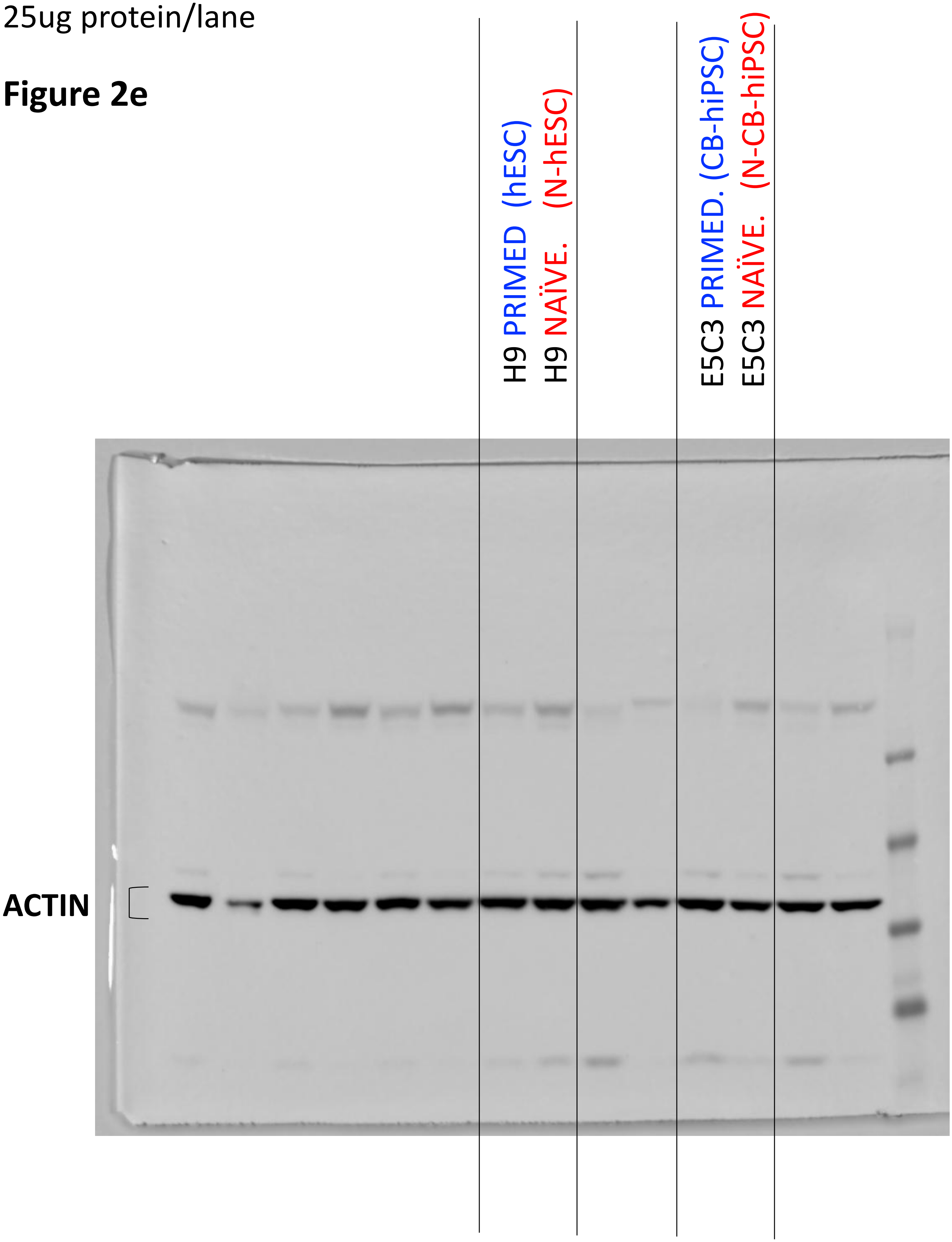

25ug protein/lane

Figure 5c

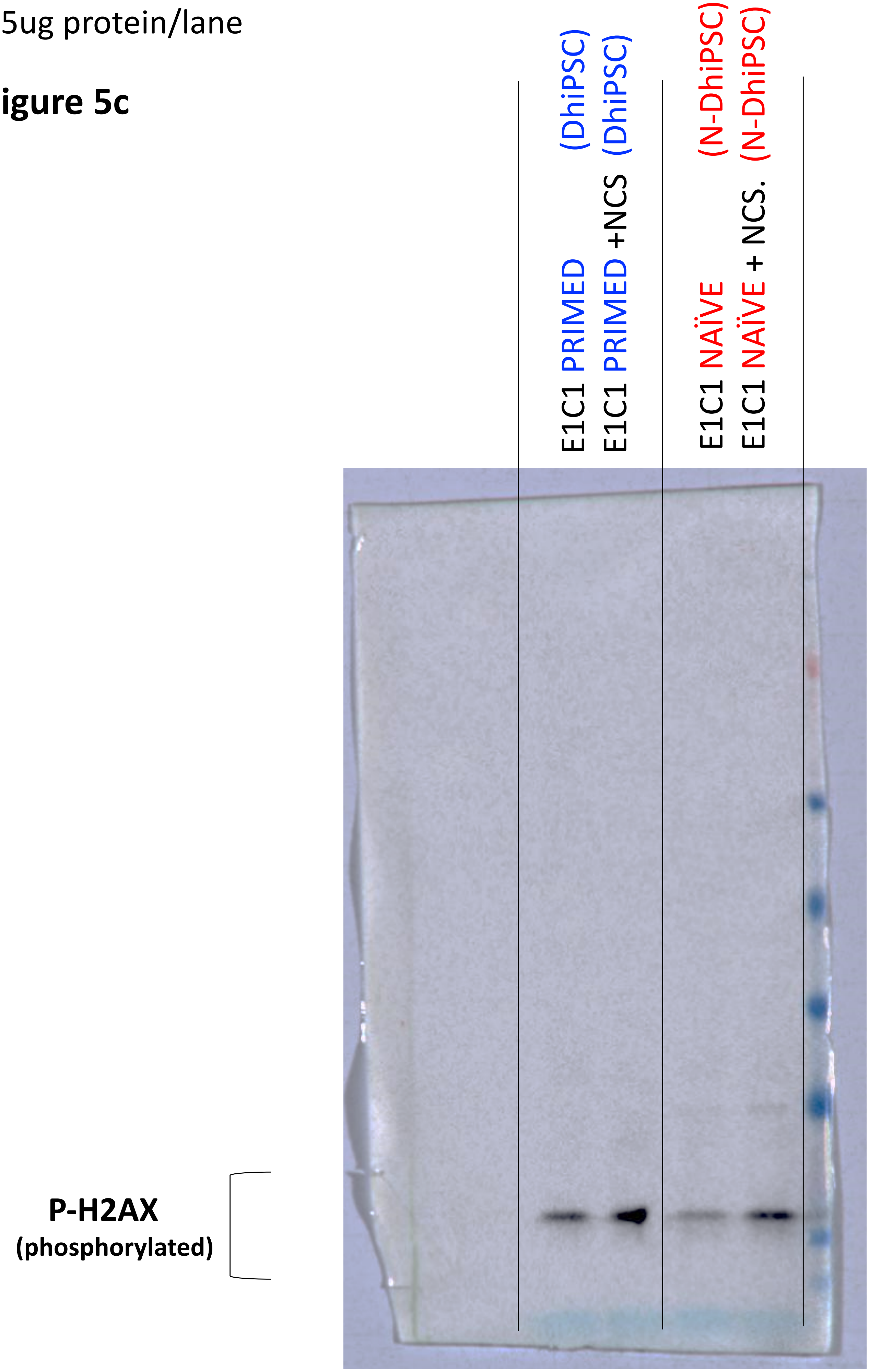

25ug protein/lane

Figure 5c

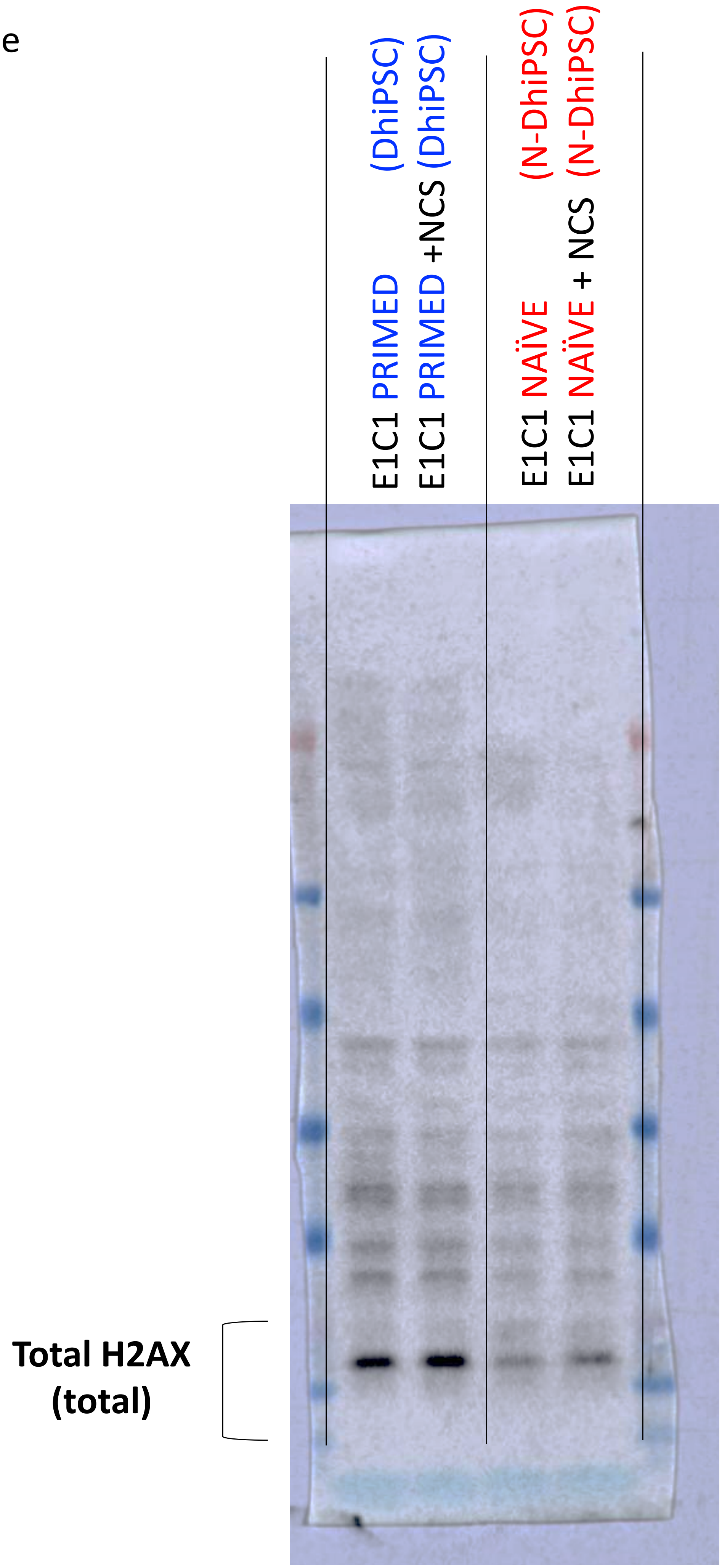

25ug protein/lane

Figure 5c

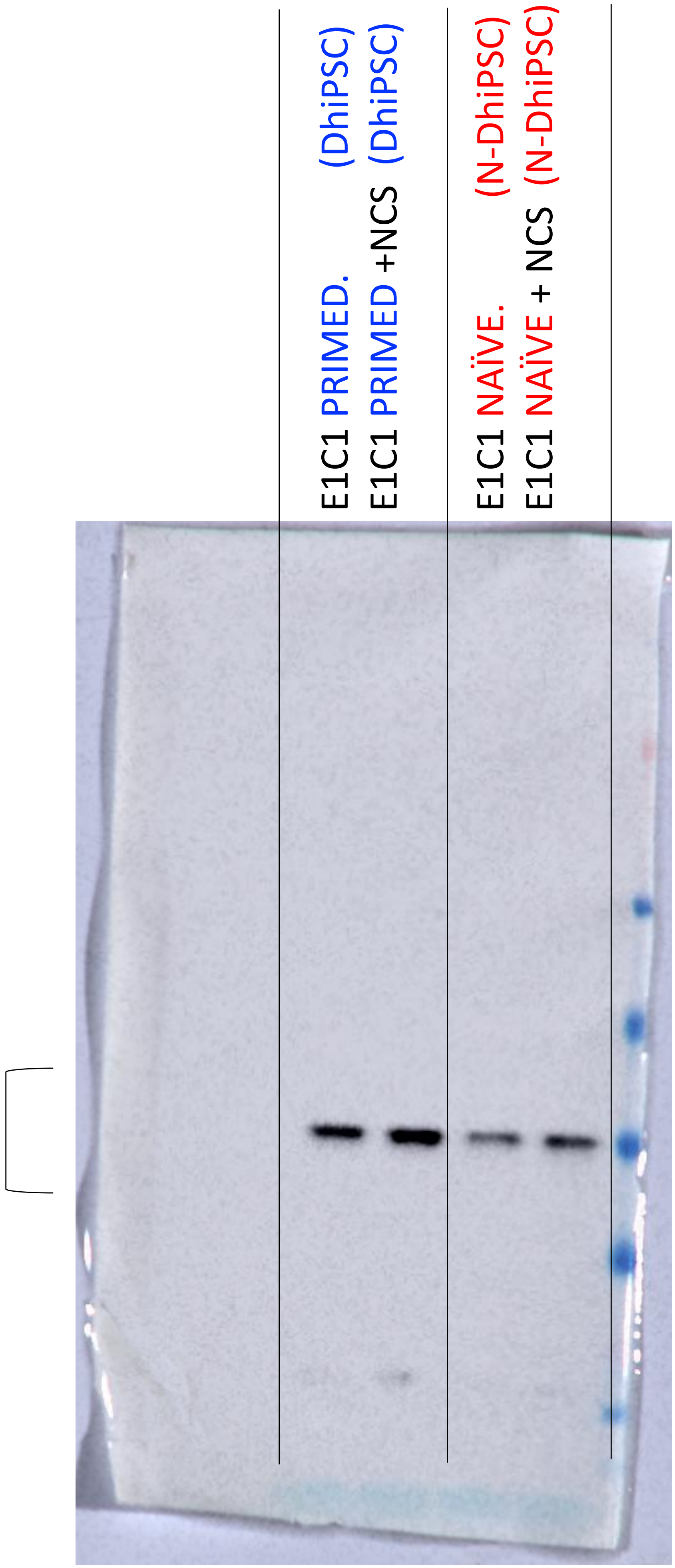

RAD51

25ug protein/lane

Figure 5c

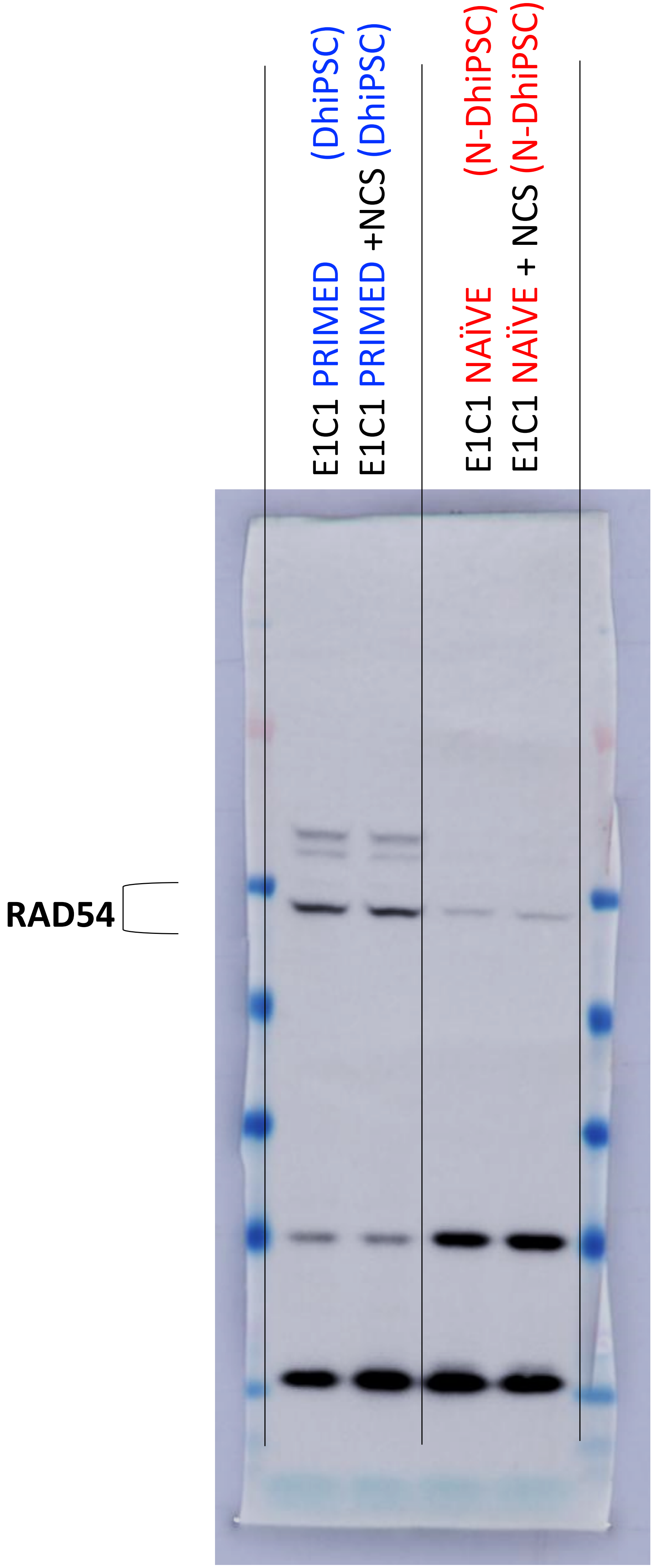

25ug protein/lane

Figure 5c

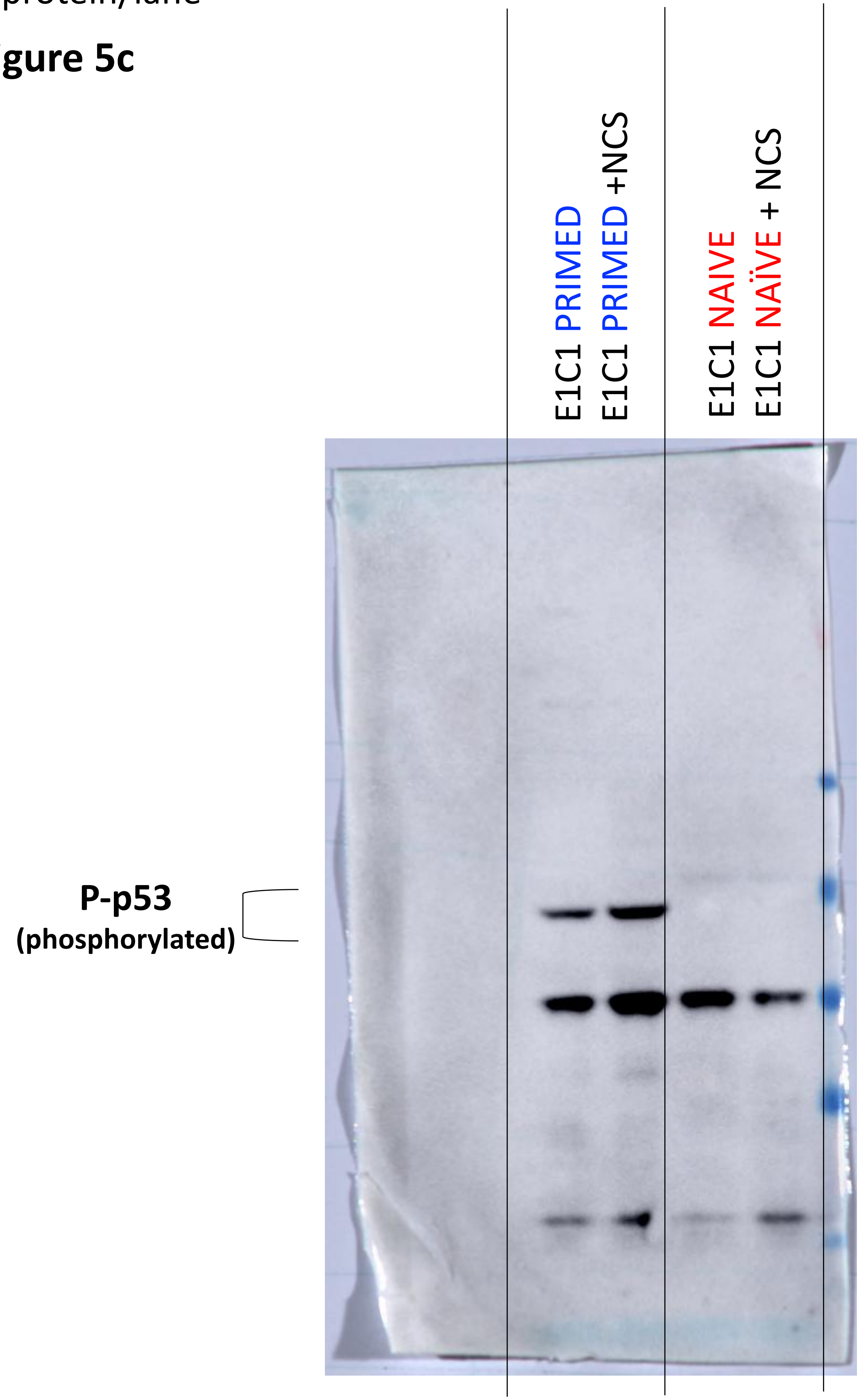

25ug protein/lane

Figure 5c

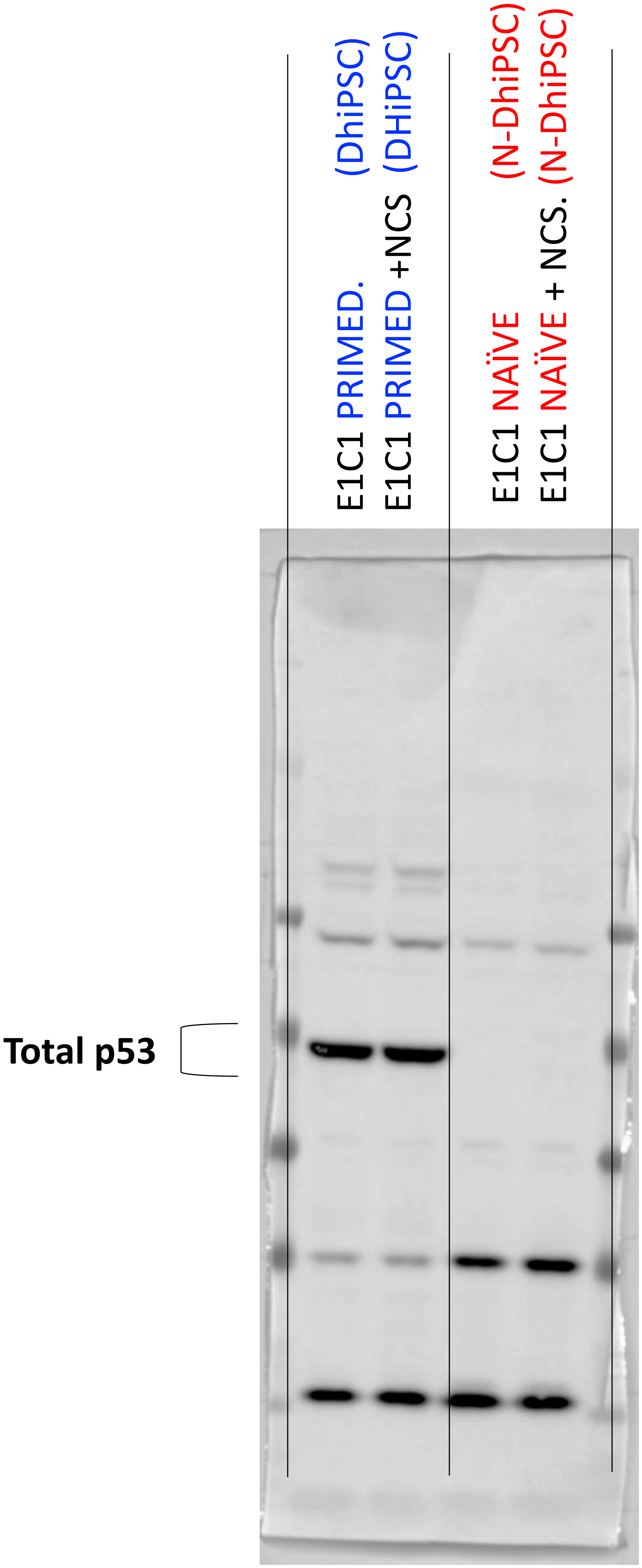

25ug protein/lane

Figure 5c

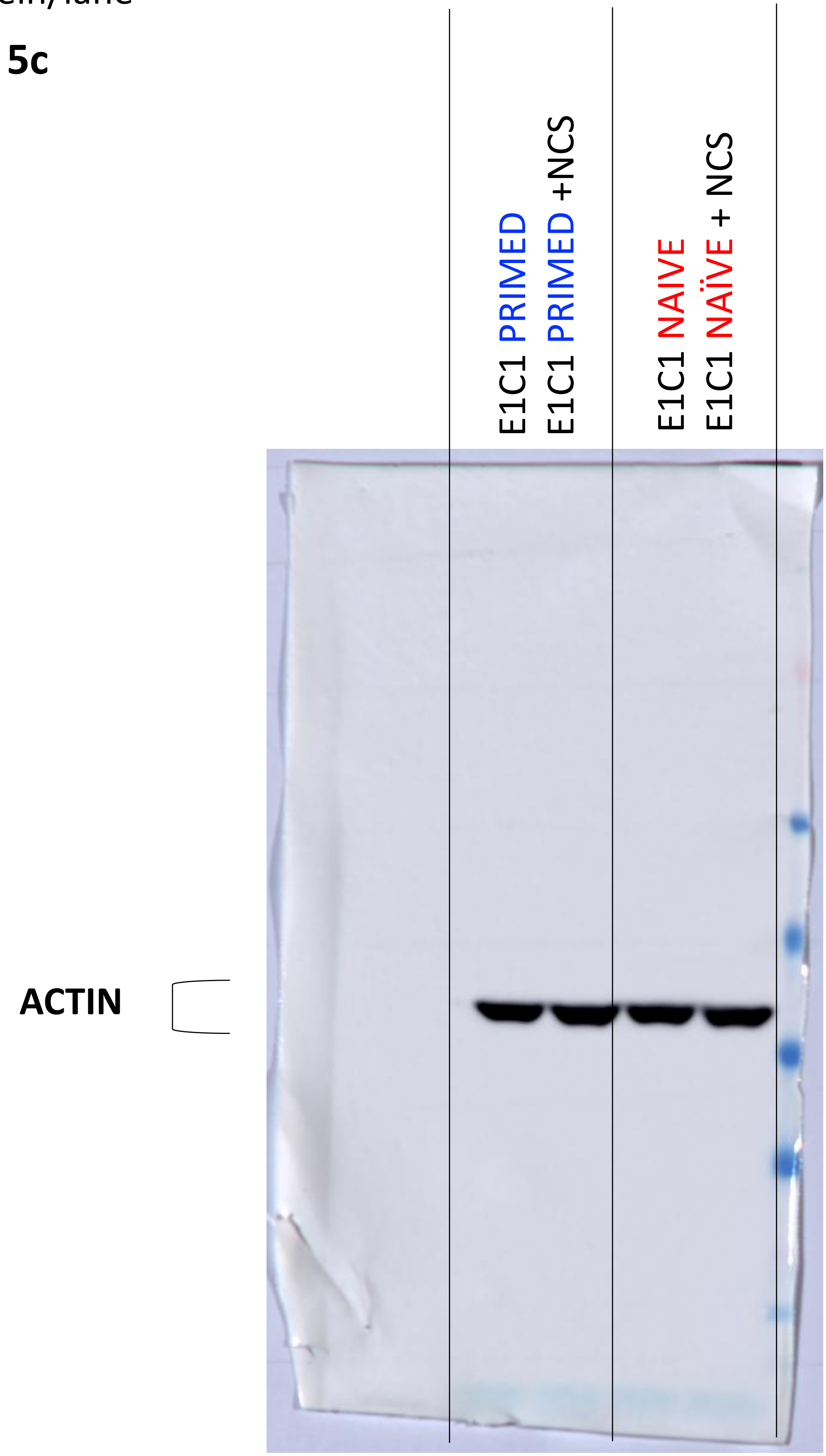

25ug protein/lane

Figure 5/Figure S5

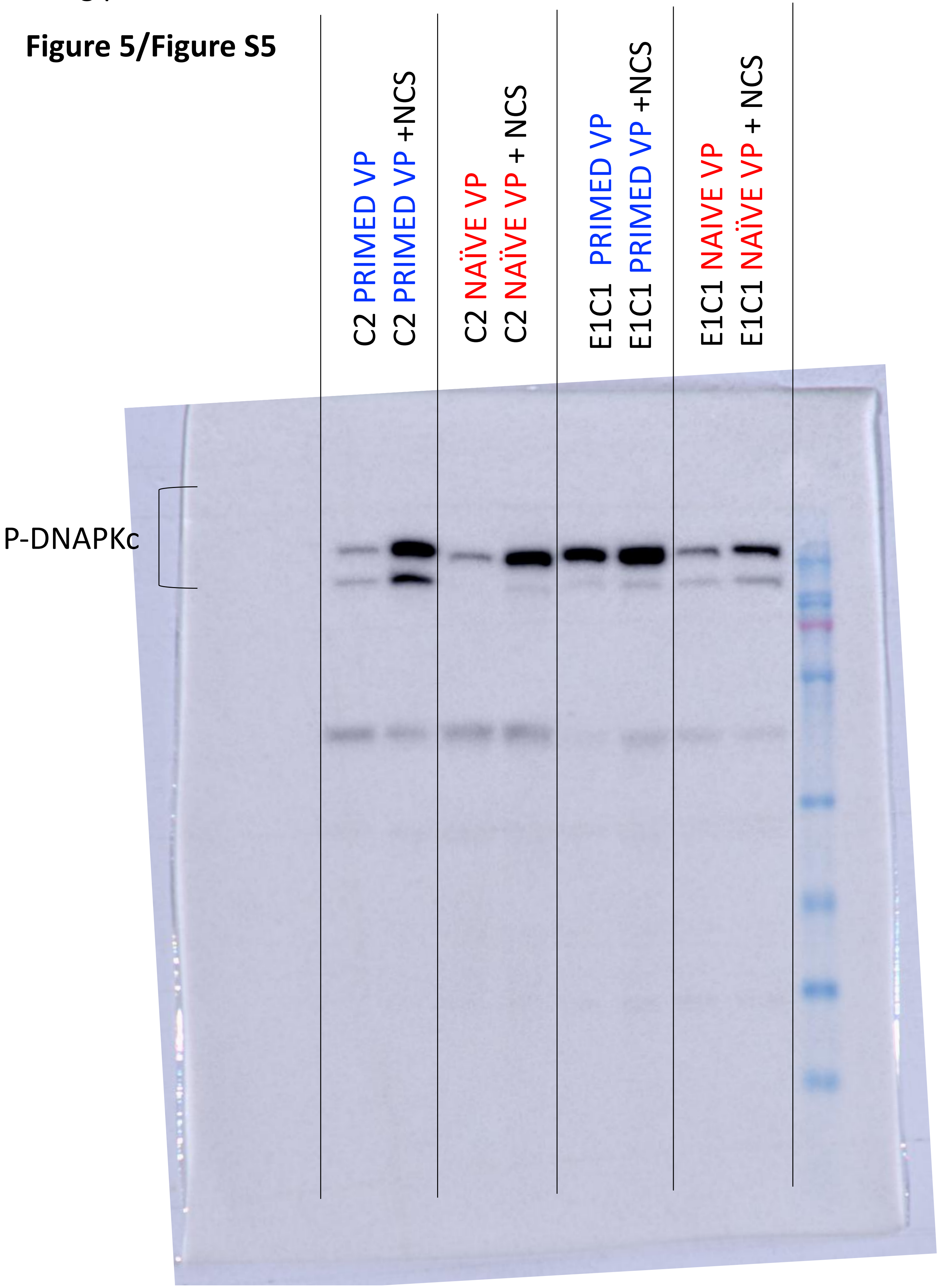

25ug protein/lane

Figure 5/Figure S5

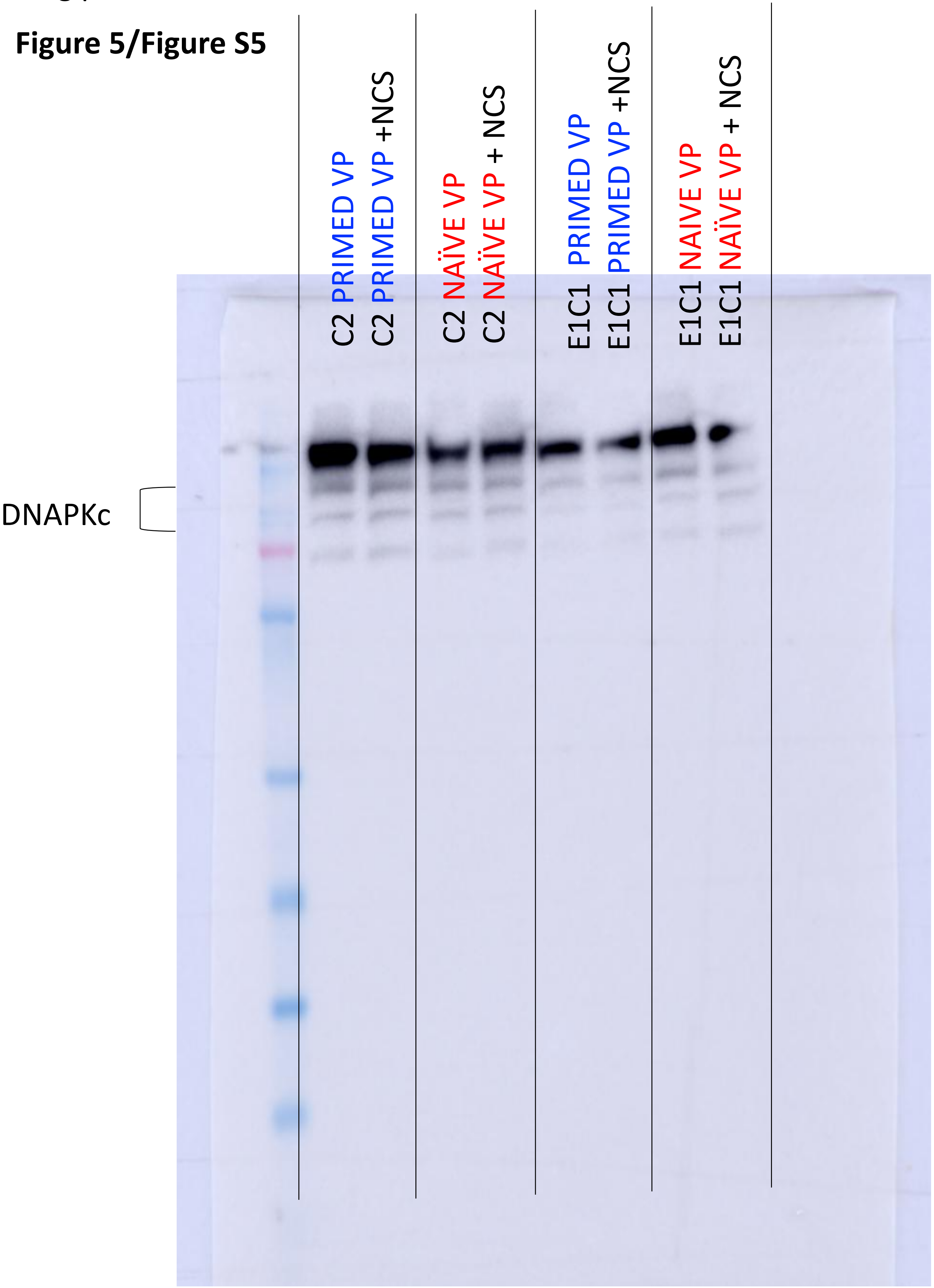

25ug protein/lane

Figure S5

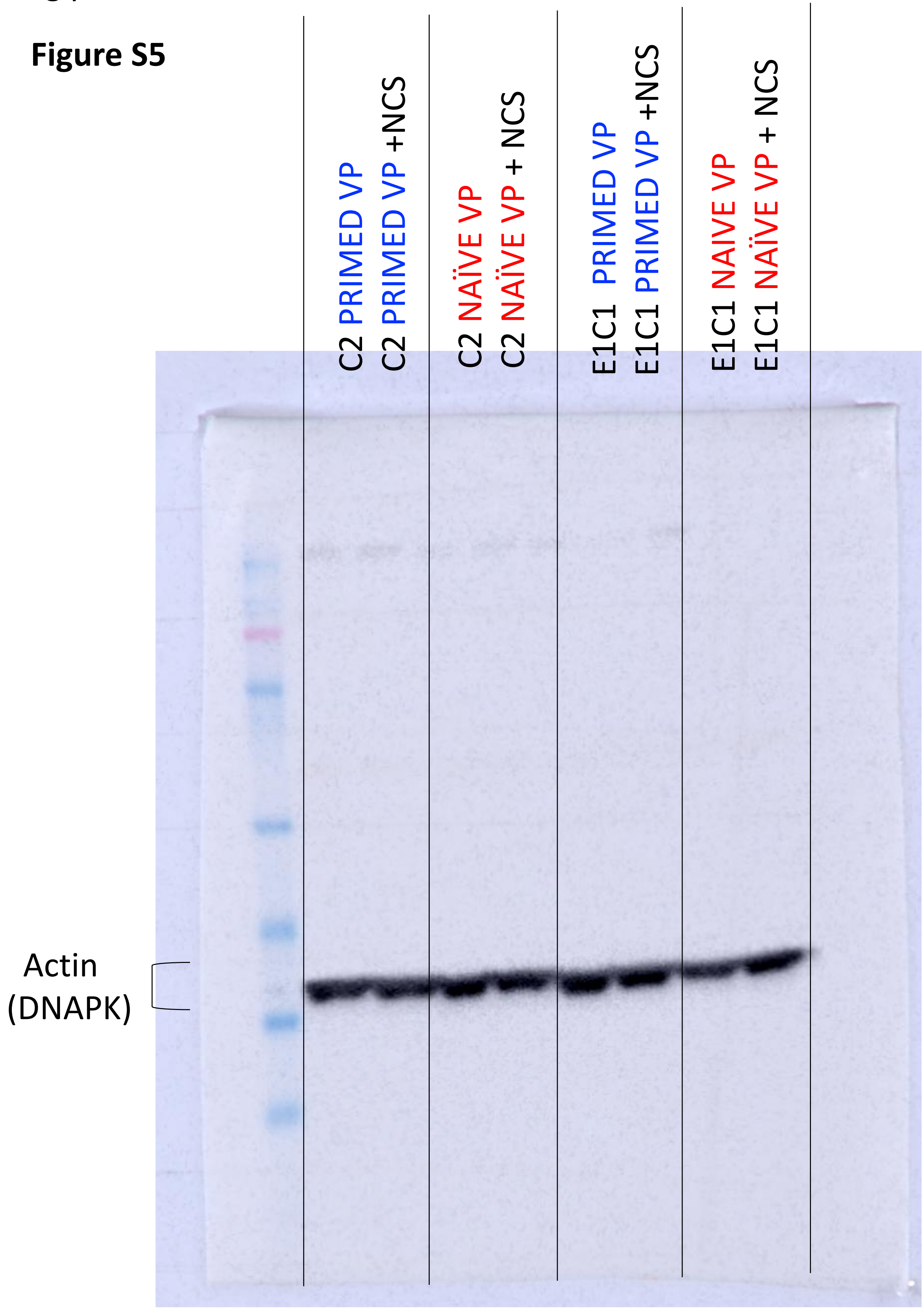

25ug protein/lane

Figure S5

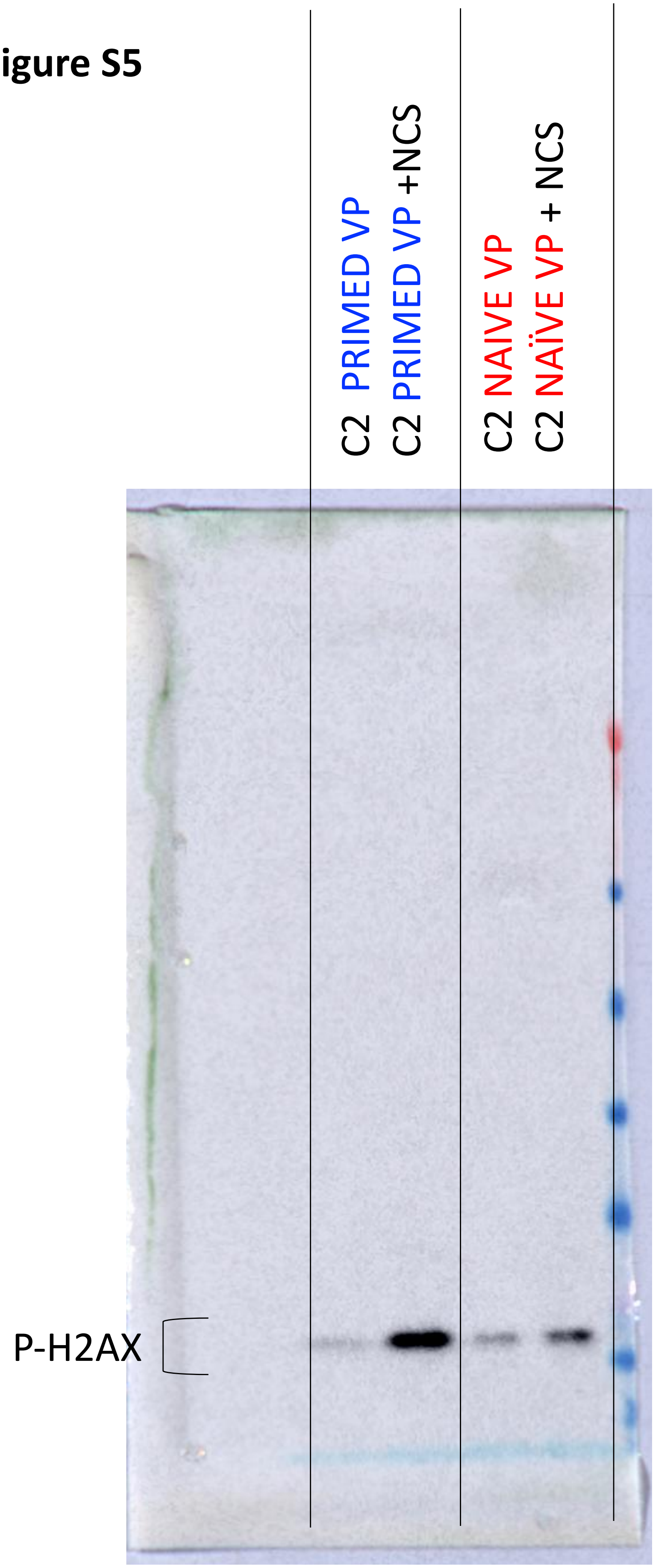

25ug protein/lane

Figure S5

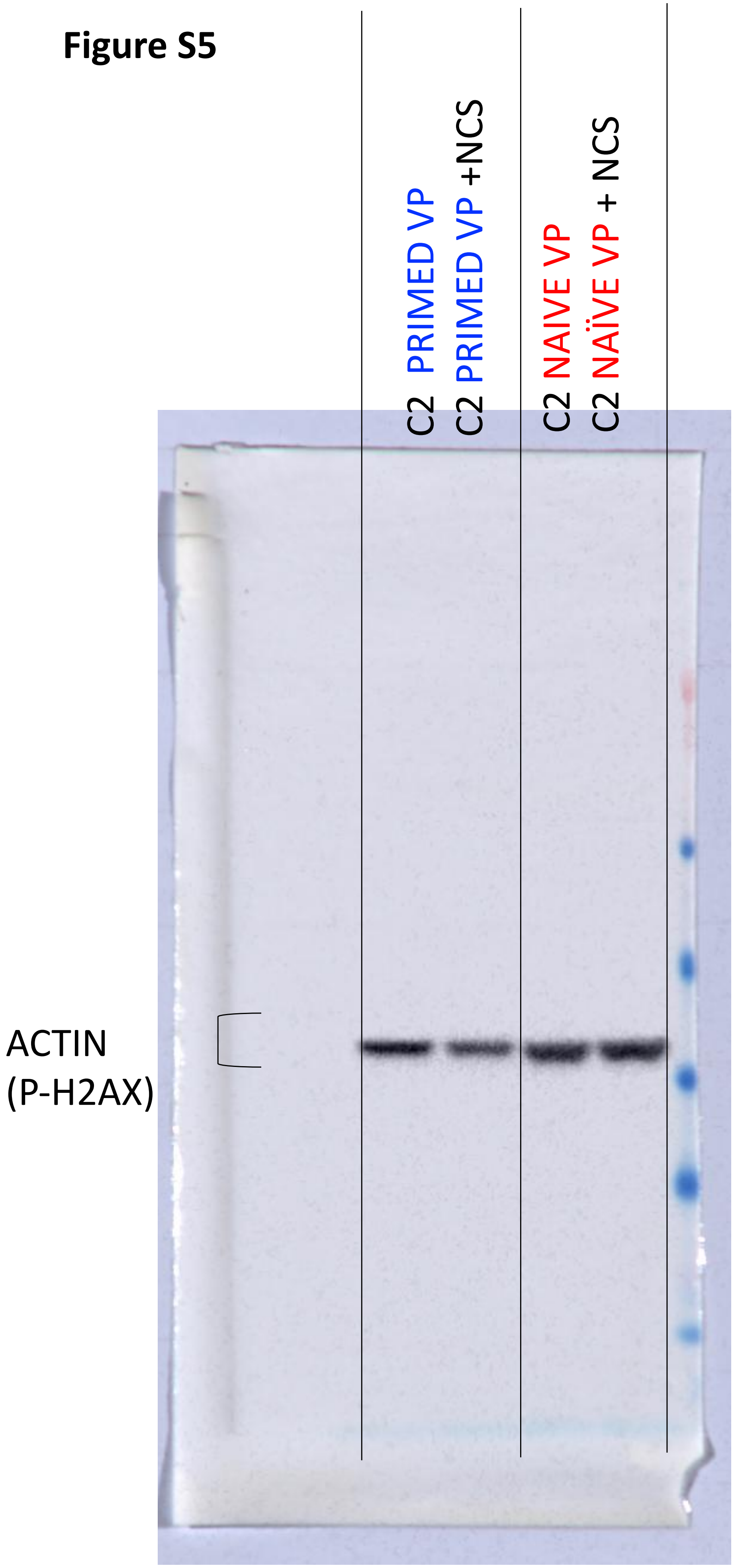

25ug protein/lane

Figure S5

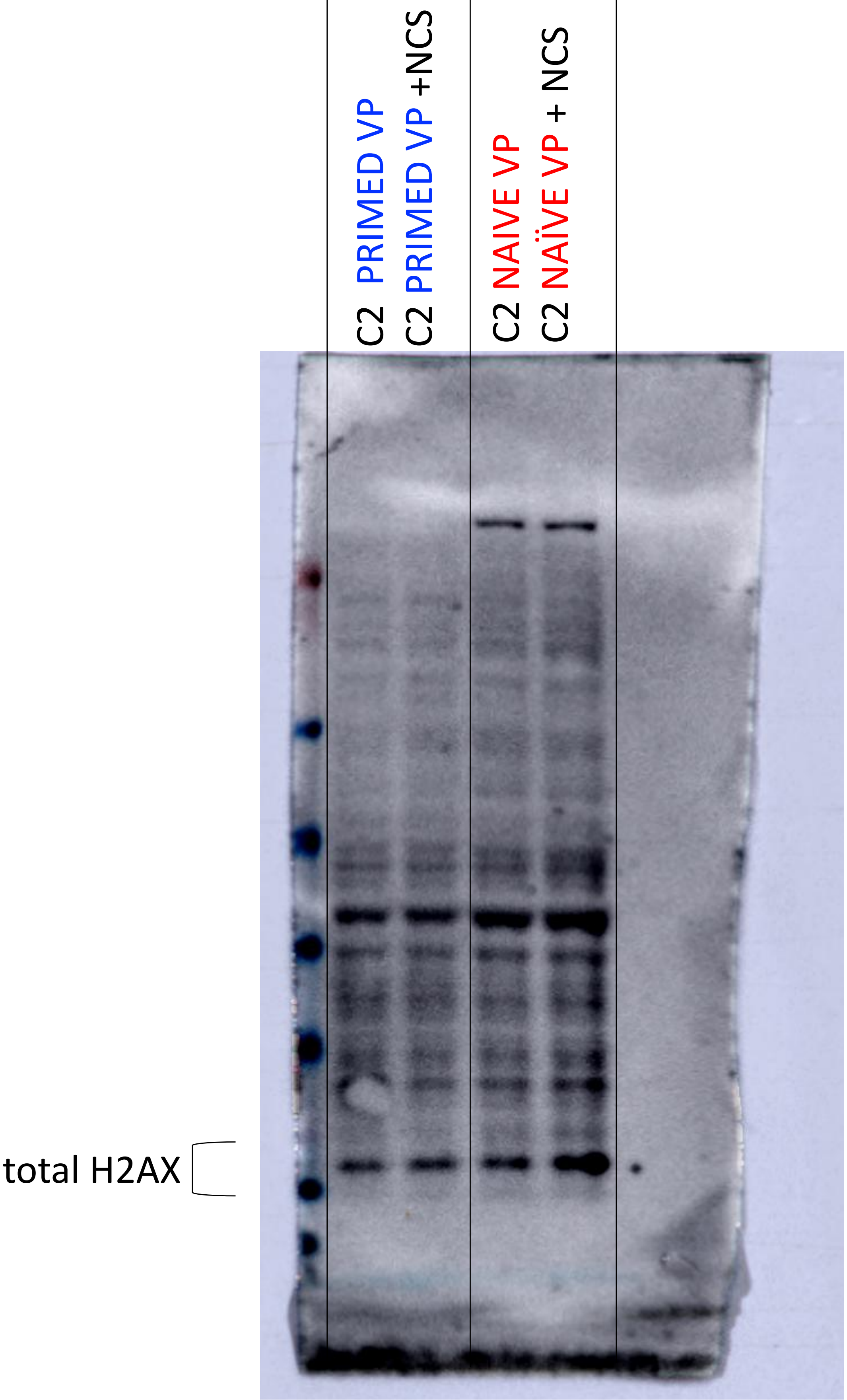

25ug protein/lane

Figure S5

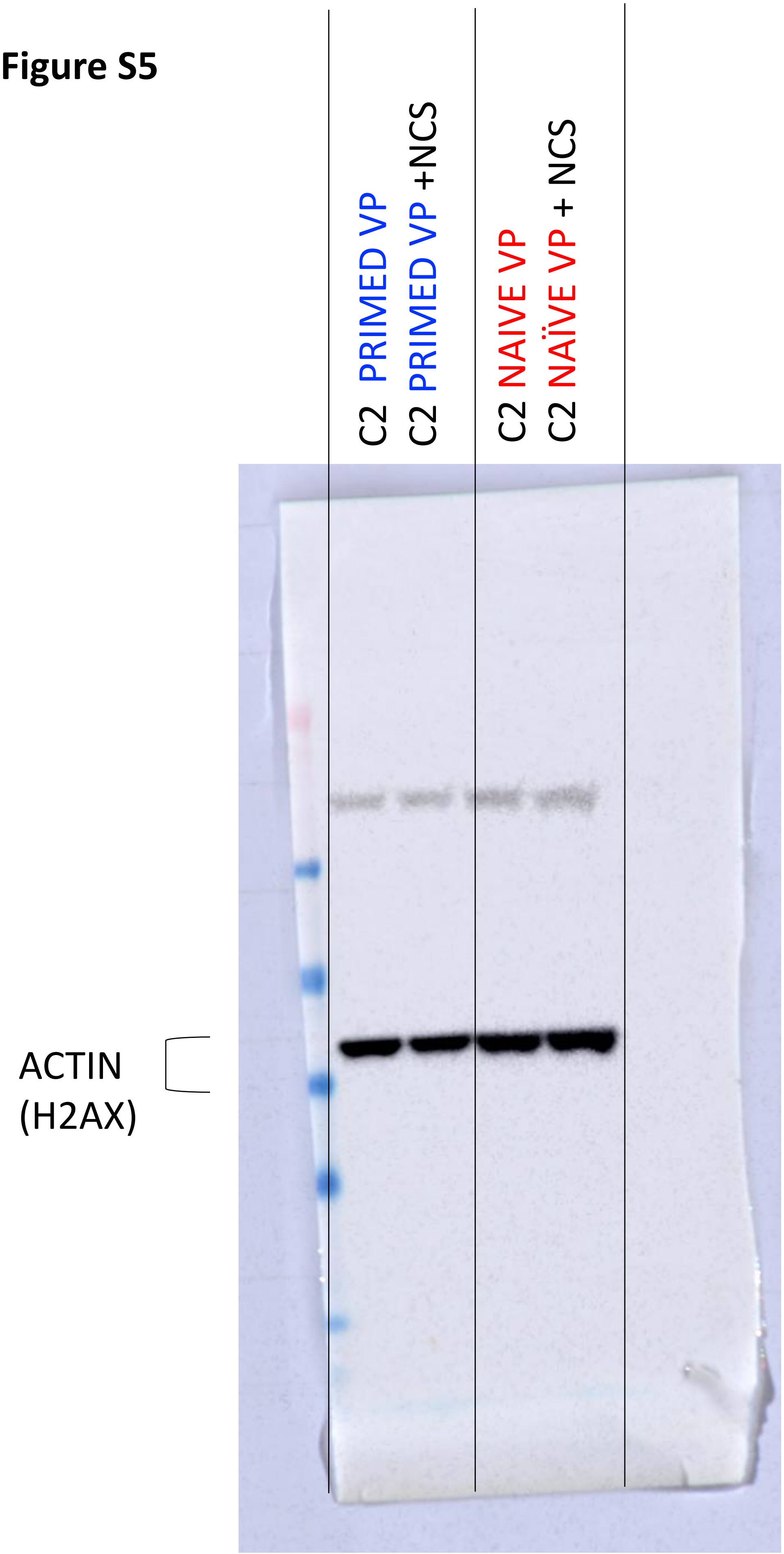

25ug protein/lane

Figure S5

E1C1 PRIMED VP

E1C1 PRIMED VP +NCS

E1C1 NAIVE VP

E1C1 NAÏVE VP + NCS

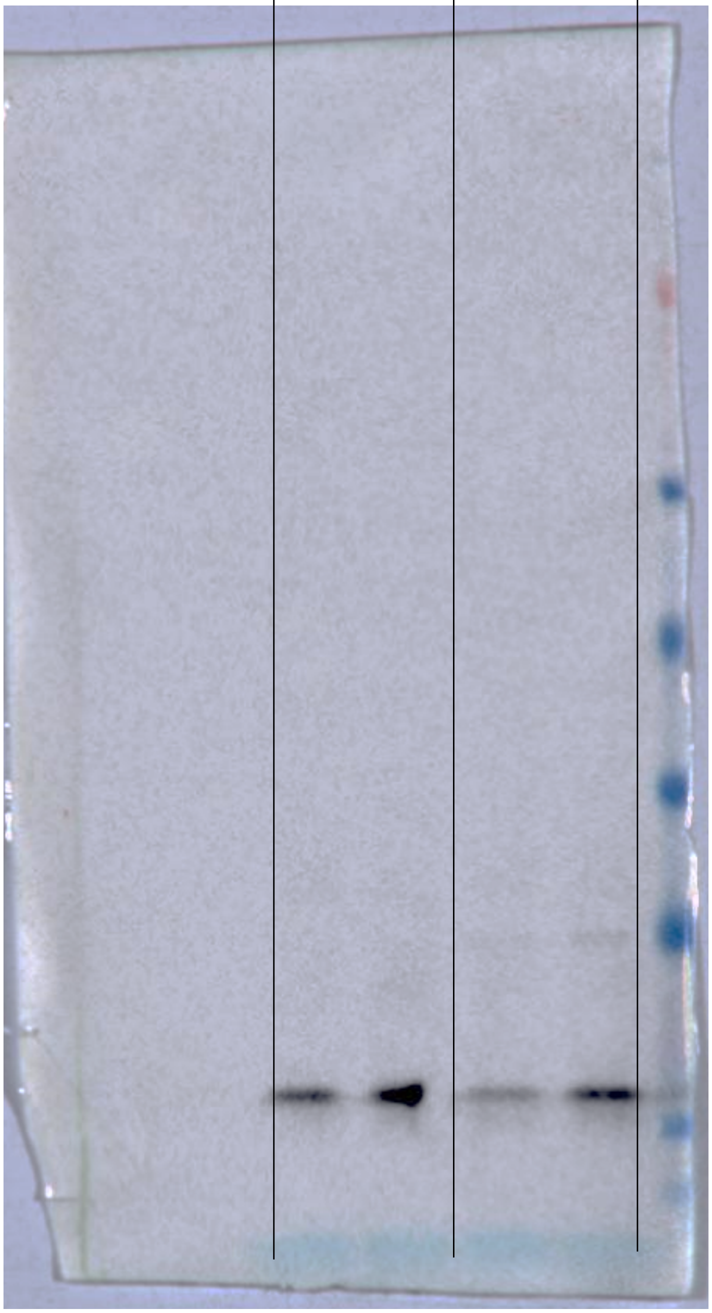

total H2AX

Figure 8a
Genomic DNA immuno-blots

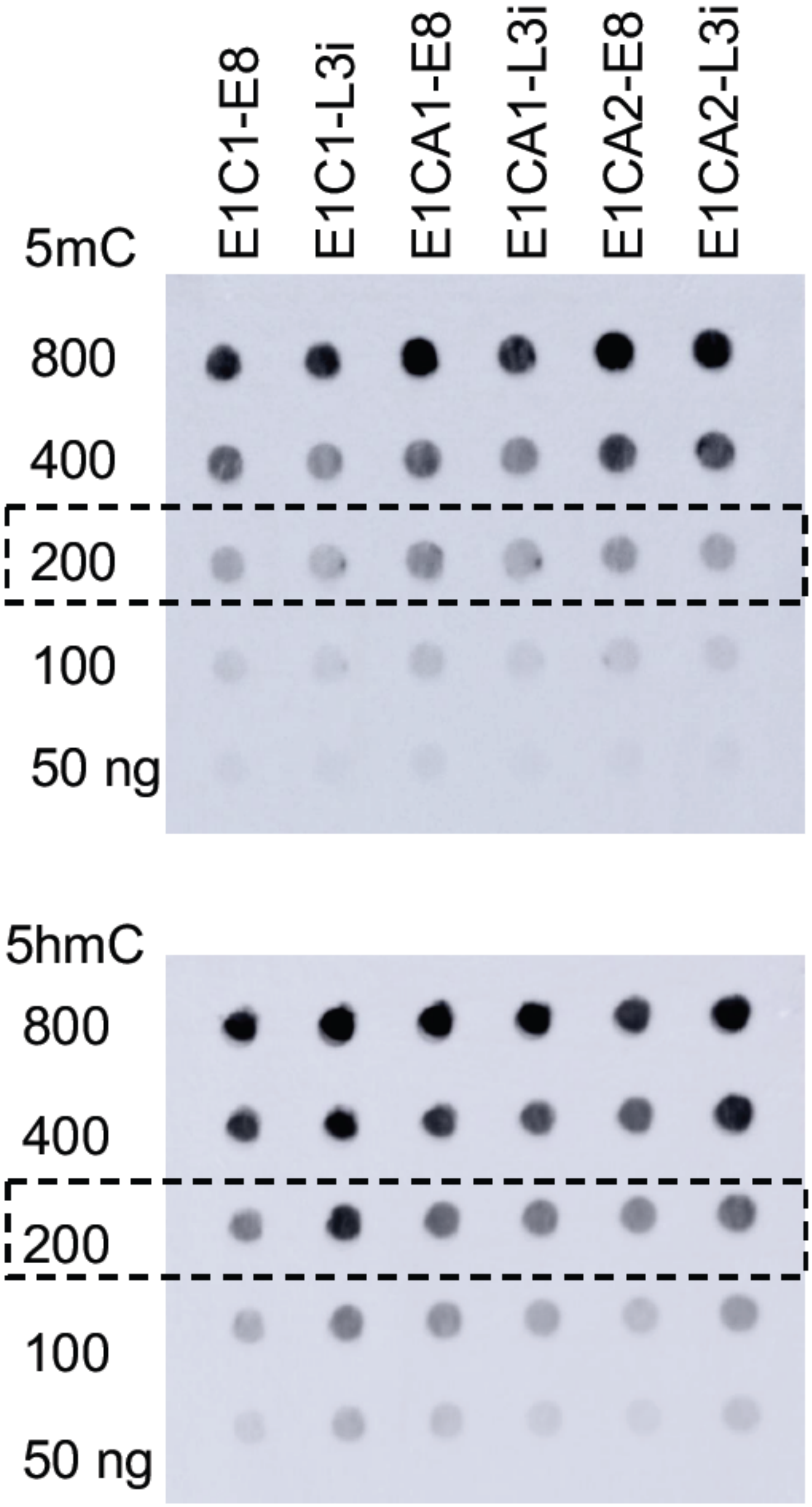

25ug protein/lane

Figure 8b

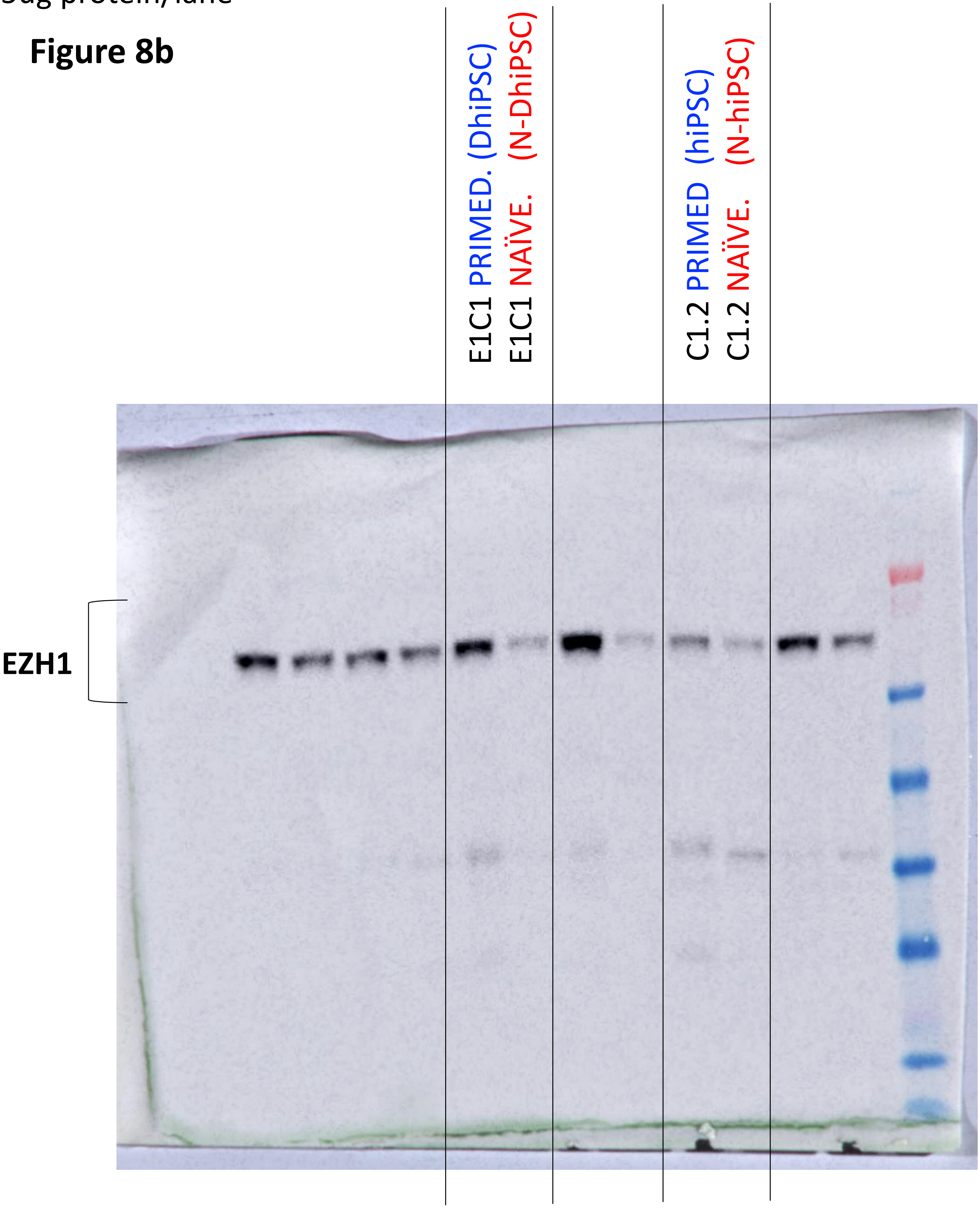

25ug protein/lane

Figure 8b

E1C1 PRIMED (DhiPSC)

E1C1 NAÏVE. (N-DhiPSC)

C1.2 PRIMED. (hiPSC)

C1.2 NAÏVE. (N-hiPSC)

EZH2

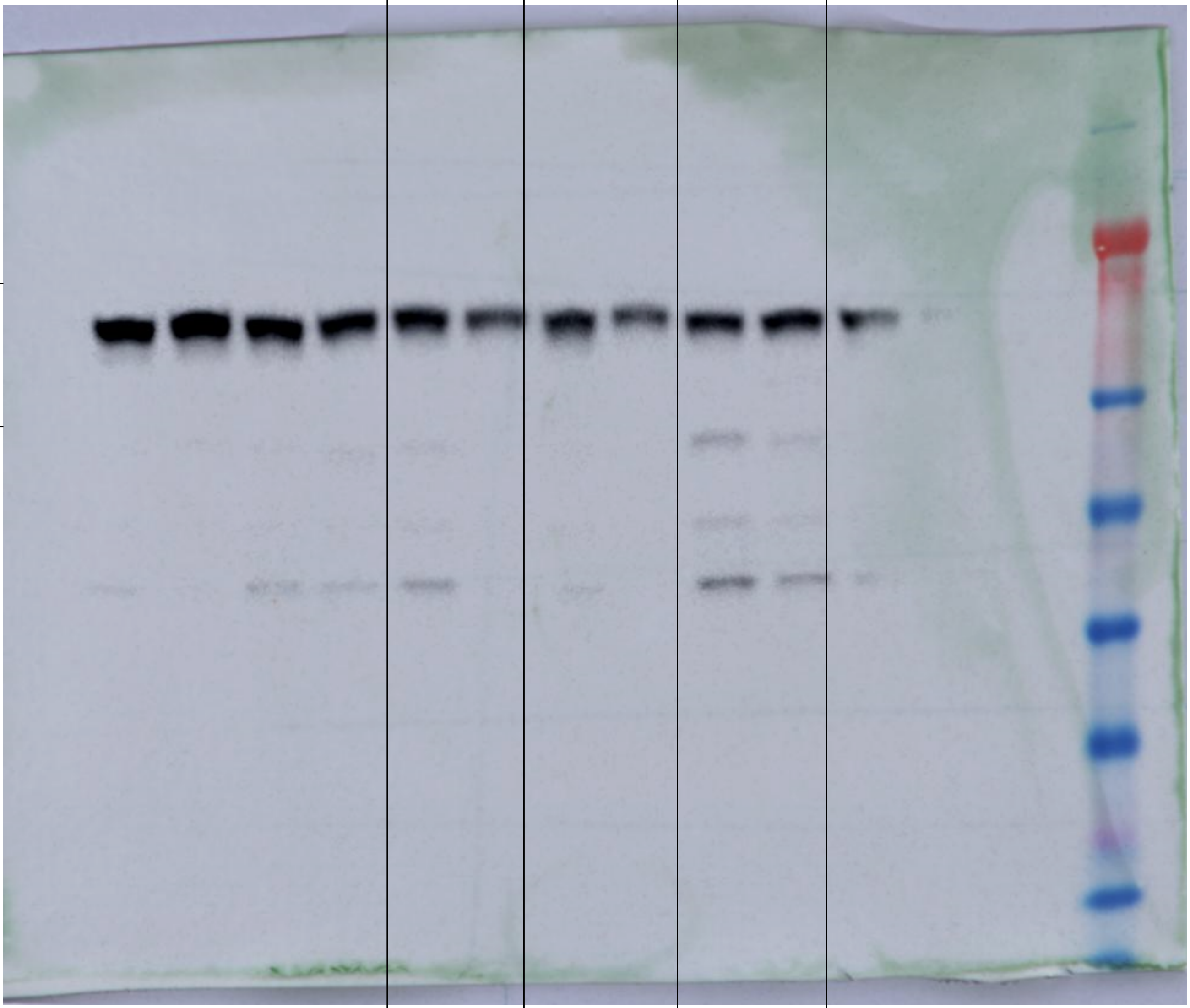

25ug protein/lane

Figure 8b

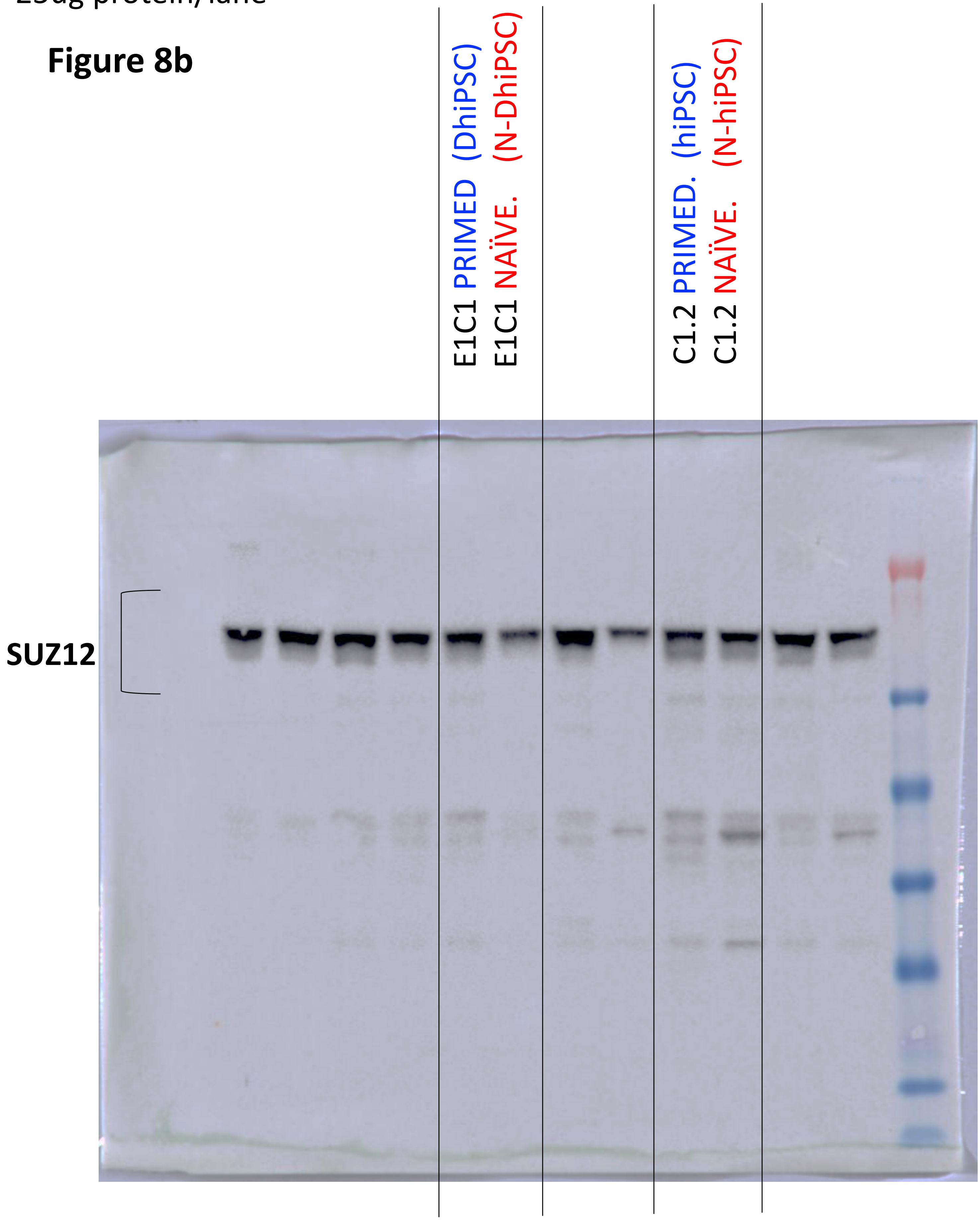

25ug protein/lane

Figure 8b

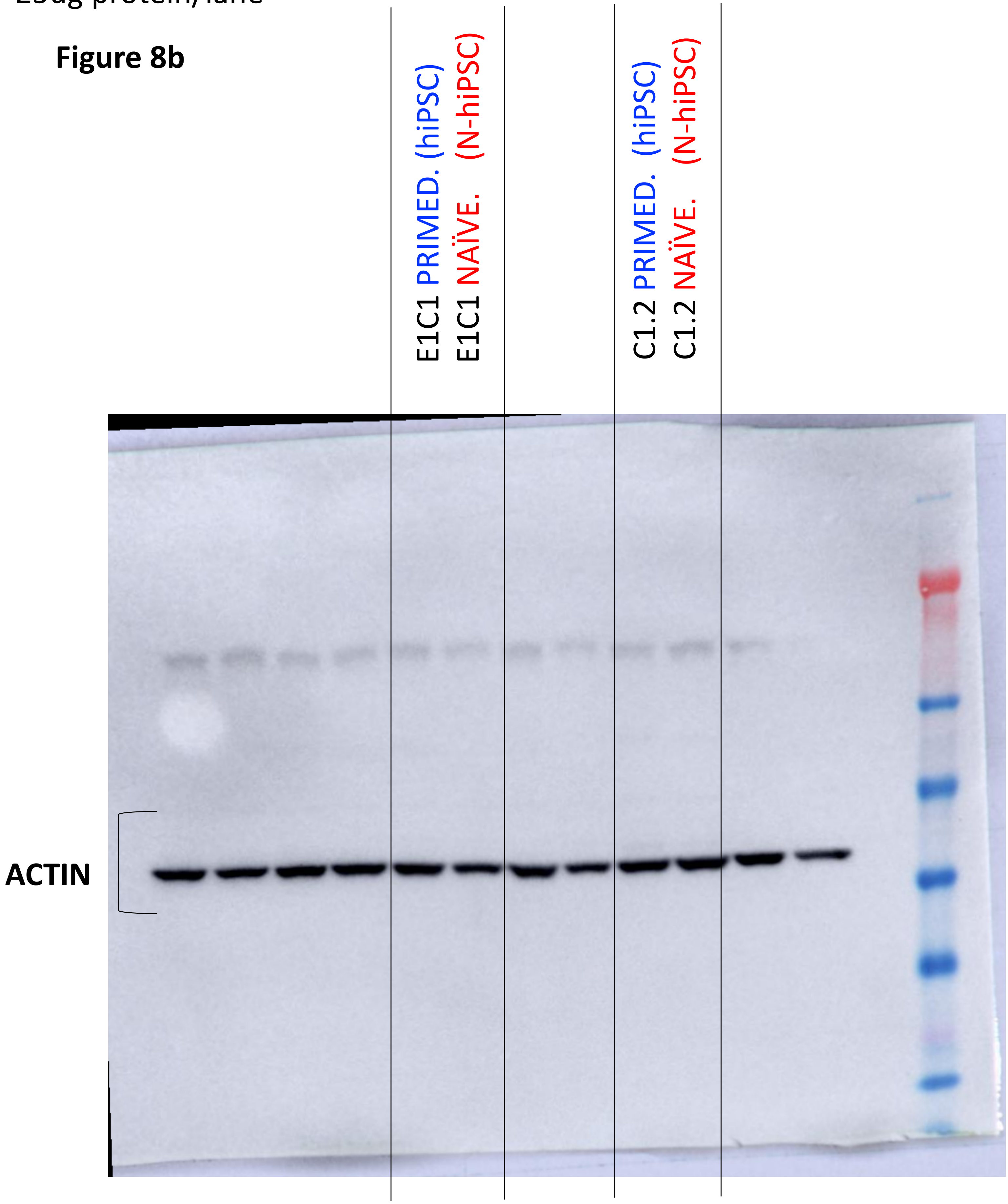

25ug protein/lane

Figure 8b

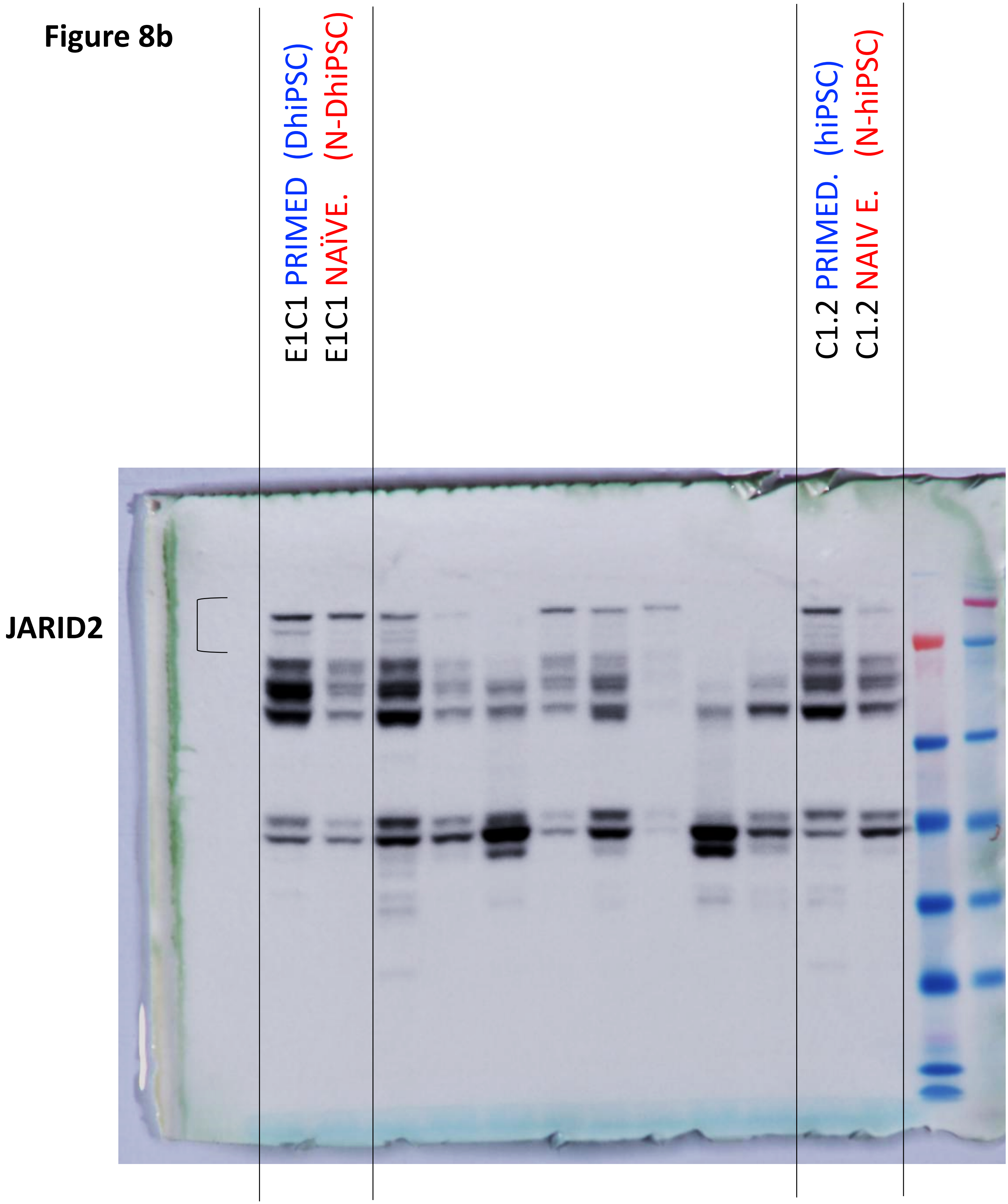

25ug protein/lane

Figure 8b

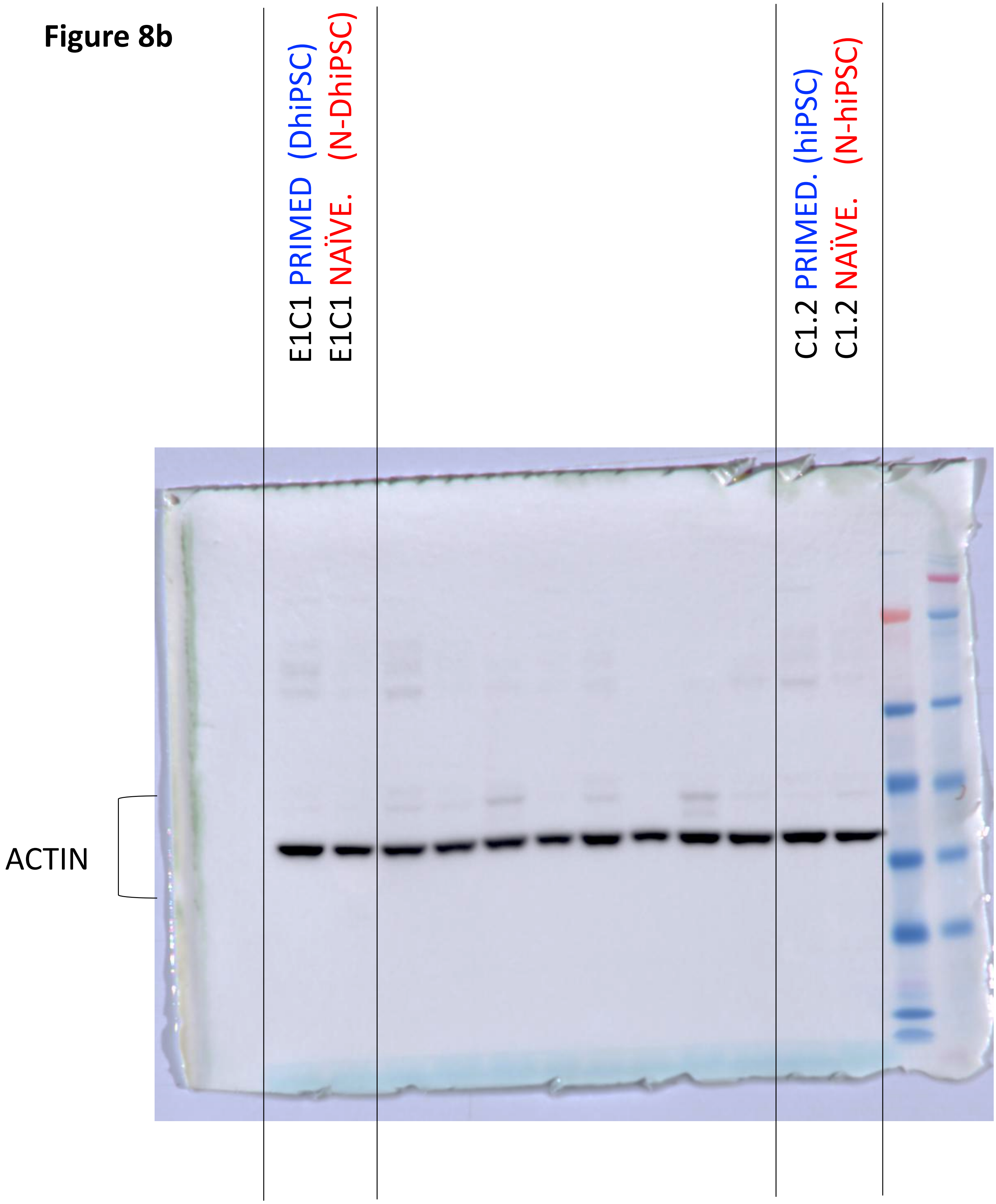

Supplement: Supplementary file 10 — Source Data [file 41467_2020_14764_MOESM10_ESM.zip › 212715_2_supp_0_q2yz4s/NCOMMS-19-18543B Source Data/Source Files/RAW WESTERN BLOTS-2.pdf]
