# Supplementary figures and images for: Vascular progenitors generated from tankyrase inhibitor-regulated naïve diabetic human iPSC potentiate efficient revascularization of ischemic retina
Source: Nat Commun. 2020 Mar 5;11:1195. doi: 10.1038/s41467-020-14764-5 (PMC7058090; doi:10.1038/s41467-020-14764-5)

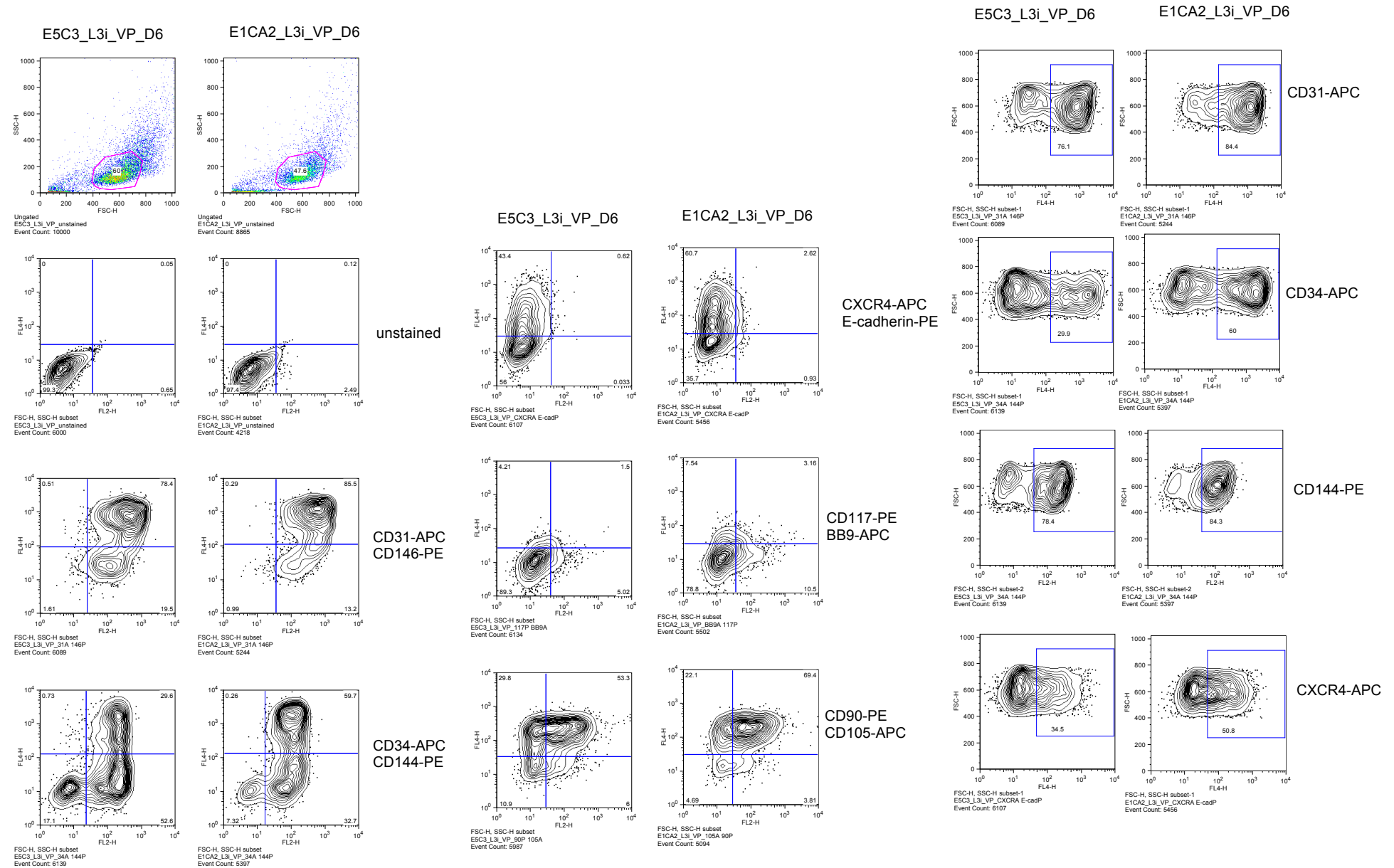

Supplement: Supplementary file 10 — Source Data [file 41467_2020_14764_MOESM10_ESM.zip › 212715_2_supp_0_q2yz4s/NCOMMS-19-18543B Source Data/Source Files/Supp Fig 4d_1-20-2017 VP.pdf]
